# A Layered, Hybrid Machine Learning Analytic Workflow for Mouse Risk Assessment Behavior

**DOI:** 10.1523/ENEURO.0335-22.2022

**Published:** 2022-01-06

**Authors:** Jinxin Wang, Paniz Karbasi, Liqiang Wang, Julian P. Meeks

**Affiliations:** 1 Department of Neuroscience, University of Rochester School of Medicine and Dentistry, Rochester, NY 14642; 2 Lyda Hill Department of Bioinformatics and BioHPC, University of Texas Southwestern Medical Center, Dallas, TX 75390

**Keywords:** hidden Markov model, machine learning, quantification of behavior, random forest, risk assessment behavior

## Abstract

Accurate and efficient quantification of animal behavior facilitates the understanding of the brain. An emerging approach within machine learning (ML) field is to combine multiple ML-based algorithms to quantify animal behavior. These so-called hybrid models have emerged because of limitations associated with supervised [e.g., random forest (RF)] and unsupervised [e.g., hidden Markov model (HMM)] ML models. For example, RF models lack temporal information across video frames, and HMM latent states are often difficult to interpret. We sought to develop a hybrid model, and did so in the context of a study of mouse risk assessment behavior. We used DeepLabCut to estimate the positions of mouse body parts. Positional features were calculated using DeepLabCut outputs and were used to train RF and HMM models with equal number of states, separately. The per-frame predictions from RF and HMM models were then passed to a second HMM model layer (“reHMM”). The outputs of the reHMM layer showed improved interpretability over the initial HMM output. Finally, we combined predictions from RF and HMM models with selected positional features to train a third HMM model (“reHMM+”). This reHMM+ layered hybrid model unveiled distinctive temporal and human-interpretable behavioral patterns. We applied this workflow to investigate risk assessment to trimethylthiazoline and snake feces odor, finding unique behavioral patterns to each that were separable from attractive and neutral stimuli. We conclude that this layered, hybrid ML workflow represents a balanced approach for improving the depth and reliability of ML classifiers in chemosensory and other behavioral contexts.

## Significance Statement

In this study, we integrate two widely-adopted machine learning (ML) models, random forest (RF) and hidden Markov model (HMM), to develop a layered, hybrid ML-based workflow. Our workflow not only overcomes the intrinsic limitations of each model alone, but also improves the depth and reliability of ML models. Implementing this analytic workflow unveils distinctive and dynamic mouse behavioral patterns to chemosensory cues in the context of mouse risk assessment behavioral experiments. This study provides an efficient and interpretable analytic strategy for the quantification of animal behavior in diverse experimental settings.

## Introduction

Behavior is the muscular output of an organism reflecting the function of the CNS (Schlinger, 2015). Accurately and efficiently quantifying animal behavior improves our knowledge of the structural and functional connectivity of the CNS underlying complex behaviors (Krakauer et al., 2017). In general, videography-based recording and manual annotation have long been a standard method for animal behavior quantification (Anderson and Perona, 2014). Recently, advancements in machine learning (ML) software has enabled tracking of animal/body parts of interest movement automatically in video recordings (Berman, 2018; Mathis et al., 2018; Pereira et al., 2019). Of these, DeepLabCut, built based on transfer learning with deep neural networks, has been widely adopted because of its efficient and intuitive framework, and supports markerless movement tracking and pose estimation (Mathis et al., 2018; Nath et al., 2019).

Regardless of the specific approach used for tracking animal positions and poses, a major challenge remains for those seeking to quantify complex animal behaviors. Typically, the multidimensional matrix of feature positions produced by DeepLabCut or other tracking methods is used as the input for additional algorithmic tools that perform dimensionality reduction, feature recognition, and ultimately a prediction of an animal’s behavioral state at each point in space and time (Dell et al., 2014; Guo et al., 2015; Machado et al., 2015). One of these tools, the random forest (RF), a versatile supervised ML algorithm, has been used in the context of human (Charles et al., 2014; Baumela, 2016) and mouse (Hong et al., 2015; Winters et al., 2022) behavioral analysis. RFs are generally able to fit complex datasets, and support multiple types of statistical analysis (Cutler et al., 2007). RF methods extract static features described by a set of variables (e.g., the matrix of feature positions) present in the inputs, and process each feature independently without influences from temporally neighboring features (Lester et al., 2005; Nath et al., 2019). This intrinsic feature of RF classifiers can produce misclassifications that may not be easy to recognize (Sok et al., 2018). For example, a mouse quickly rearing during exploration may be indistinguishable from a long instance of standing or defending against an aggressor. This limitation of RF classifiers limits their utility for complex, temporally-evolving components of behavior.

Behavior intrinsically involves a sequential pattern of movements, and behavioral events occur in probabilistic relationships with the environment (Fountain et al., 2020). Hidden Markov models (HMMs), stochastic time-series models, infer that an observed event sequence is driven by a series of transitions between hidden states (Leos‐Barajas et al., 2017). Once trained and validated, two types of information can be obtained from HMMs: the sequence of predicted hidden states and model transition probabilities between those states. HMMs have been extensively applied in multiple fields, such as speech recognition, bioinformatics, as well as the analysis of animal behavior (Findley et al., 2021; Jiang, 2021; Mor et al., 2021).

HMMs support behavioral classification tasks in many scenarios, but also have drawbacks (Glennie et al., 2022; Ruiz-Suarez et al., 2022). HMMs are unsupervised and parametric, but do not necessarily produce results aligned to the hypotheses being tested (Bicego et al., 2006). Thus, one must explore and optimize many parameters to produce results that relate to the hypotheses being tested by the human experimenters (Ahmadian Ramaki et al., 2018). For instance, the determination of the optimal number of hidden states is difficult to determine, often requiring subjective evaluation of results to avoid overgeneralization or fragmentation, both of which are barriers to interpretation and hypothesis testing (Deo, 2015; Wuest et al., 2016; Pohle et al., 2017; H. Liu and Shima, 2021). Despite their multiple advantages for quantifying temporally complex behaviors, the drawbacks of HMMs can limit their utility in behavioral neuroscience.

**Figure 1.**
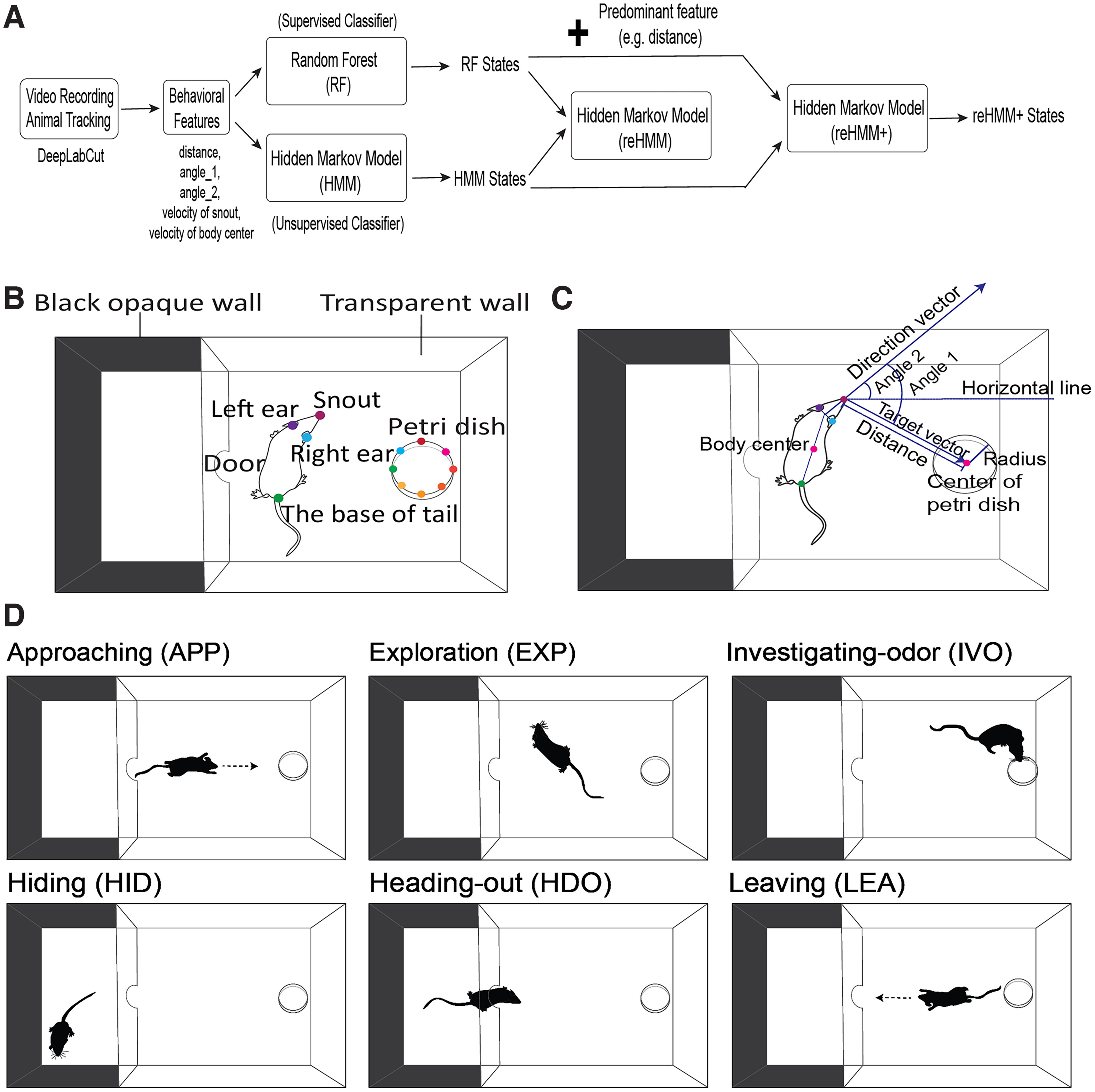
***A***, Architecture of analytic workflow and behavioral experiment overview. Mouse movement and body parts are tracked by using DeepLabCut software. Behavioral features (e.g., distance, angle_1, angle_2, and velocities of snout and body center) are calculated using DeepLabCut outputs and are used to train a random forest (RF) and a hidden Markov model (HMM) with equal numbers of states, separately. The per-frame predictions from RF and HMM are passed to a second HMM layer (reHMM). The predictions from RF and HMM plus predominate positional features are used to train a third HMM (“reHMM+”). ***B***, Diagram of the behavioral test arena and DeepLabCut labeling. Mice are tracked by an overhead camera during video recording. For DeepLabCut labeling, four mouse body parts (snout, left ear, right ear, and the base of tail) are labeled. Eight labels equally spaced on a circle are used to label the Petri dish. ***C*,** Graphical representation of derivative features. ***D***, Ethogram including six behavioral states for mouse risk assessment behavior, including approaching (APP), exploration (EXP), investigating-odor (IVO), hiding (HID), heading-out (HDO), and leaving (LEA).

Given that both supervised and unsupervised ML algorithms have their own merits and demerits, several methods have employed both supervised and unsupervised ML methods, creating so-called hybrid ML models. Hybrid ML models attempt to use supervised and unsupervised methods (e.g., RFs and HMMs) to compensate for demerits of each process when used alone. In the present study, we used positional features obtained from DeepLabCut as inputs for a layered, RF-HMM hybrid ML analytic workflow (Fig. 1*A*). We employed this workflow to reveal that mice display fear-like response to 2,4,5-Trimethylthiazole (TMT) with a reduced tendency of approaching odor and a rapid-and-short investigative pattern. Also, we uncovered that predatory chemosensory cues resulted in an increased behavioral trend of risk assessment in mice. We found that the resulting layered, hybrid workflow produces rich, interpretable models that support hypothesis testing in chemosensory and other complex behavioral contexts.

## Materials and Methods

### Mice

All animal procedures were performed in accordance with the University of Texas Southwestern Medical Center or University of Rochester animal care committee's regulations. Wild-type C57Bl/6 mice were obtained from The Jackson Laboratory (stock #000651) or the Mouse Breeding Core at University of Texas Southwestern Medical Center. All mice aged 8–15 weeks were kept with 12/12 h light/dark cycle (lights on from noon until midnight). Mice were given *ad libitum* access to food and water. Throughout the manuscript, the number of animals is described in text and figure legends.

### Behavioral test setup

Mouse risk assessment behavior was investigated in a customized two-chamber arena (Fig. 1*B*). The testing arena consisted of a small rectangular chamber with three opaque black walls [6” (L) × 8” (W) × 8” (H)] and a large rectangular chamber with three transparent walls [11” (L) × 8” (W) × 8” (H)]. The two chambers were divided by a transparent wall with an open door. Mice could freely cross two chambers through the door. Mice are tracked by an overhead FLIR Blackfly camera (BFS-U3-16S2C-CS, USB 3.1) at 60 frames per second (FPS).

### DeepLabCut tracking

DeepLabCut (2.2.0.3) was used to track all points of interest (Mathis et al., 2018; Nath et al., 2019), including four labels in the mouse body (snout, right ear, left ear, and the base of tail) and eight labels equally spaced in the Petri dish (Fig. 1*B*). To create a robust network, we added a different number of new videos (frames) to retrain the existing network in each behavioral test because of slight discrepancies in experimental conditions, including lighting, testing arena, animal, and background. Cumulatively, a total of 9621 labeled frames were used to train ResNet-50-based neural networks with default parameters for 200,000–800,000 iterations. For all labeled frames, 80% was used for network training, whereas the remaining 20% was used for network evaluation. If the test error with p-cutoff was around five pixels (image size was 1160 × 716 pixels; 1 mm ≈ 2 pixels), this network was then used to analyze videos recorded in similar experimental conditions. To account for residual low-confidence tracking data and swapped or missing labels, we applied a rolling median filter to tracking data. Before finalization, labeled videos created by DeepLabCut were manually spot-checked for accuracy.

### Behavioral features and ethogram

The output of DeepLabCut was in the form of *x/y* pixel coordinates of each label. We calculated several derivative features from DeepLabCut outputs that matched the experimental design (Fig. 1*C*). Specifically, we calculated the distance between the mouse snout and the center of the Petri dish (“distance”). “Direction vector” was the vector from the midpoint of two ears toward the mouse snout. “Target vector” was the vector from the mouse snout to the center of the Petri dish. “Angle_1” was the angle between the direction vector and the target vector. “Angle_2” was the angle between the direction vector and a horizontal line (*x*-axis). “Velocity of snout” was the instantaneous velocity (in pixels) of the snout from one frame to the next. “Body center” was the midpoint between the base of tail and the midpoint of two ears. “Velocity of body center” was the instantaneous velocity (in pixels) of the body center from one frame to the next.

To study mouse risk assessment behavior, we defined a simple ethogram consisting six behavioral states, which was modified from the previous study (Dielenberg and McGregor, 2001; Fig. 1*D*). Hiding (HID) was the state that the mouse stayed in the smaller, dark rectangular chamber. Heading-out (HDO) was the state that the mouse body crossed the door and toward the larger clear rectangular chamber. Approaching (APP) was the state that the mouse moved toward the Petri dish directly from the small rectangular chamber. Leaving (LEA) was the state that the mouse moved toward the small, dark rectangular chamber directly after sniffing the Petri dish. Investigating-odor (IVO) was the state that the mouse snout was located within the diameter of the Petri dish. Exploration (EXP) was all mouse behavior except for IVO in the large rectangular chamber.

### Odor stimuli preparation

2,4,5-Trimethylthiazole (TMT) was purchased from Sigma-Aldrich. TMT was diluted 1:9 (10%) with mineral oil (Saito et al., 2017). Female mouse urine was collected as previously described and stored at −80°C in a freezer (Holekamp et al., 2008; “Female_urine”). Snake fecal samples were collected from the Department of Herpetology at the Dallas Zoo. Fecal samples of inland taipan (*Oxyuranus microlepidotus*) and black mamba (*Dendroaspis polylepis*) were used in the behavior test. Snake feces was directly placed in the Petri dish (“Feces”). For the preparation of snake fecal extracts, inland taipan feces particles (5 g) were placed in 50 ml of distilled water (dH_2_O). The feces mixture was homogenized by vortexing for 2 min and placed on ice on an orbital shaker overnight. On the second day, the feces mixture was homogenized by vortexing for 2 min and then sequentially subjected to two steps of centrifugations (10 min at 2400 × *g* at 4°C and 30 min at 2800 × *g* 4°C). The supernatant from two centrifugations was pooled in collection tubes (“s_unfiltered”) and filtered with a 0.22 μm filter (“s_filtered”). Fecal solids (“solid”) were also kept and stored at −80°C. Pure water (dH_2_O) was used as the control odor (“control”).

### Risk assessment behavior test

All behavioral tests were performed during the dark cycle under dim red light. For odor presentation, 3D-printed fake fecal particles (1 cm in length with an ellipse shape) were immersed in odor solutions overnight before the experiment day. Fake fecal particles (*n* = 5–7) were placed in the Petri dish and kept wet during the experiment. To avoid cross-contamination, one Petri dish was used only for one odor. In all behavioral tests, mice were exposed to each odor for 5 min.

**Figure 2.**
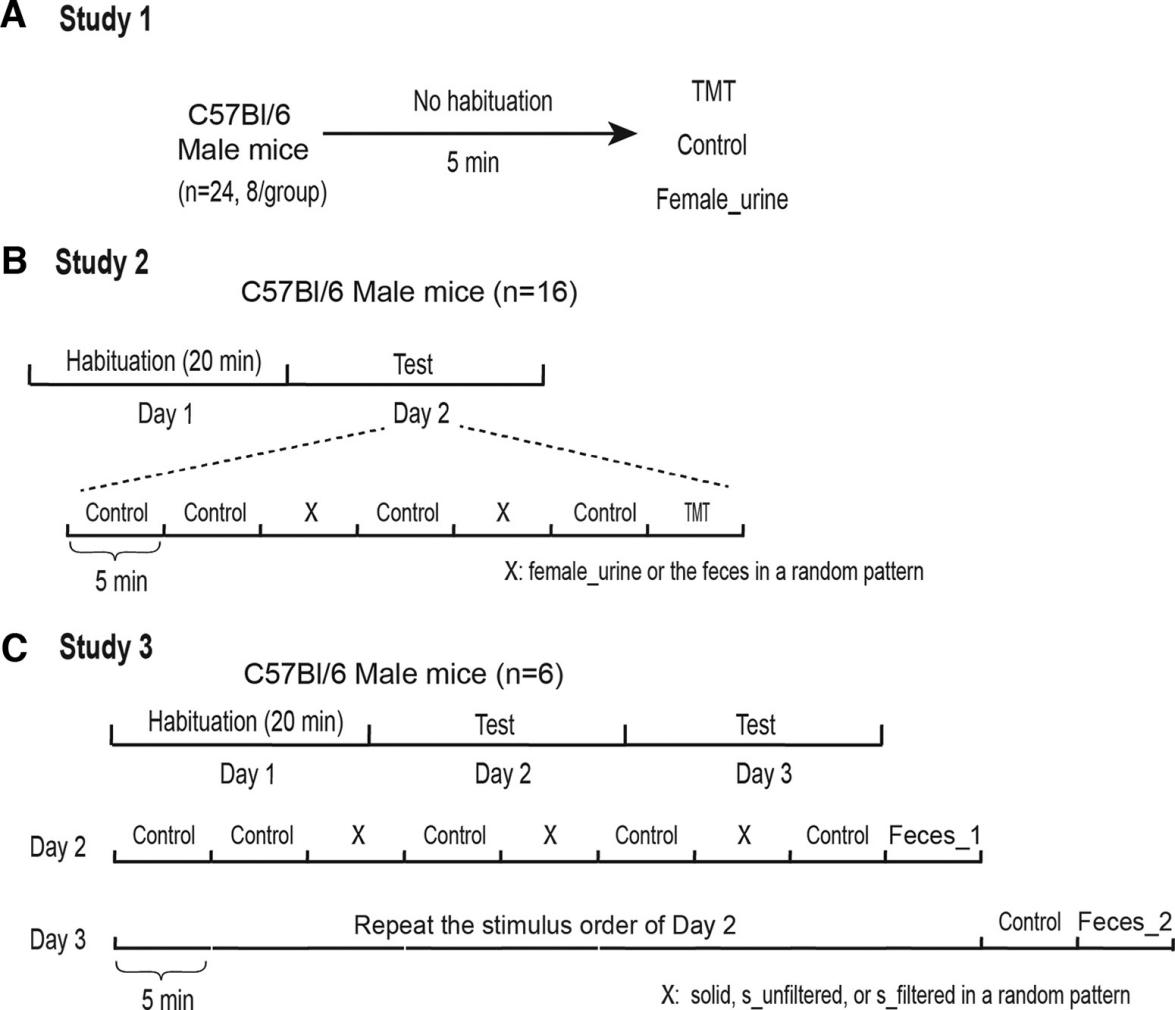
Schematic diagrams of experimental design of risk assessment behavior test. ***A***, Experimental design of Study 1. ***B***, Experimental design of Study 2. ***C***, Experimental design of Study 3.

For the first study (Fig. 2*A*), 24 C57Bl/6 male mice were randomly divided into three groups (*n* = 8 for each group) and exposed to control, TMT, and female_urine, respectively. Without habituation, mice were placed in the test arena for 5 min. Behavioral tests were performed on three consecutive days and only one group (one odor) was tested each day.

For the second study (Fig. 2*B*), 16 C57Bl/6 male mice were used. Control, TMT, female_urine, and the feces were used as odor treatments. One day before the experimental test, mice were placed in the test arena for 20 min to habituate to the novel environment. On the testing day, mice were sequentially exposed to odors in sequence as “control-control-X-control-X-control-TMT,” in which X represented either female_urine or the feces in a random pattern. Because of its well-known fear-inducing effect, TMT was always the last odor delivered to mice. Interspersed control treatments were intended to reduce the potential for stimulus order effects. The second of the initial control treatments was used as the “control” sample for statistical purposes.

For the third study (Fig. 2*C*), six C57Bl/6 male mice were used. Control, inland taipan feces (Feces_1), black mamba feces (Feces_2), solid, s_unfiltered, and s_filtered stimuli were used as odor treatments. One day before the experimental test, mice were placed in the test arena for 20 min to habituate to the novel environment. Then, mice were subjected to 2-d testing. On the first testing day, mice were sequentially exposed to odors in sequence as “control-control-X-control-X-control-X-control-Feces_1,” in which X was one of solid, s_unfiltered, and s_filtered in a random pattern. The testing on the second day exactly repeated the stimulus order of the first day, except for an additional “control-Feces_2” that was added at the very end. As above, interspersed control treatments were interspersed between odor treatments, and the second of the initial control treatments was used as the “control” for statistical analysis.

### Random forest

RF classification was performed by using Python (3.8.12) in the Conda environment (4.10.3). The RF classifier (sklearn.ensemble.RandomForestClassifier) was loaded from the Scikit-learn library (Pedregosa et al., 2011). The hyperparameter n_estimators was defined as 50 and all others were default values. As the ground truth (GT) in this study, six behavioral states (HID, APP, HDO, LEA, EXP, and IVO) were manually annotated frame-by-frame in 12 videos. Five behavioral features (distance, angle_1, angle_2, velocity of snout, and velocity of body center) extracted from 12 manual annotated videos (a total of 149,258 frames) were used to train the RF model. For all frames, 80% was used for the RF classifier training, whereas the remaining 20% was used for model evaluation. The RF performance was evaluated by 10-fold cross-validation (sklearn.model_selection.KFold). Feature importance analysis was conducted by using the module (sklearn.feature_selection. SelectFromModel) from the Scikit-learn library. To further improve the performance of the RF, behavioral feature data were smoothed using a rolling median filter (window sizes = 5, 10, 15, 30, and 60) from the Pandas library (pandas.Series.rolling). After data smoothing, 30 behavioral features (five original features plus 25 smoothed features) were used to train a new RF model. The same approaches mentioned above were used to evaluate the performance of the new RF model.

### Hidden Markov models

We tested several Hidden Markov Model (HMM) based approaches, including auto-regressive HMMs, Hidden Semi-Markov Models, and Hierarchical HMMs using the extensions available at https://github.com/lindermanlab/ssm). We found that the model (hmm.GaussianHMM) from the hmmlearn Python library (https://github.com/hmmlearn/hmmlearn) produced the most useful outputs in our experimental conditions. The hyperparameters of the HMM model was as follows: covariance_type=“diag,” n_iter = 100, verbose=True, random_state = 0. “n_components” representing the number of hidden states was adjusted as needed. The same five behavioral features used to train the RF were also fed to train the HMM model. The outcomes of the HMM were evaluated by comparing it with the GT and manually checking the plots of the behavioral features of each HMM hidden state. For HMM model optimization, Expectation-Maximization (EM) algorithm and Bayesian Information Criterion (BIC) were used as criteria to determine the “n_components” parameter. For the second HMM (“reHMM”) and third HMM (“reHMM+”), the training data sequences (RF classification, HMM classification, and distance) were compressed by replacing with the most frequent element (RF classification and HMM classification)/median (distance) in every 15 frames. “n_components” of reHMM and reHMM+ models were six, and other hyperparameters were identical to HMM. The classifications of RF and HMM were fed to train the reHMM. The classifications of RF and HMM and distance were used to train the reHMM+. The same evaluation methods for HMM were used to evaluate the performance of the reHMM and reHMM+.

#### Linear discriminant analysis (LDA)

In our reHMM+ workflow, many measurement metrics were generated to quantify mouse behavior, such as state occupancy, state transition probability, movement distance, etc. Thus, we used a supervised classifier linear discriminant analysis (LDA) to make an overall comparison of behavioral responses across all animals. The LDA classifier (sklearn.discriminant_analysis.LinearDiscriminantAnalysis) was loaded from the Scikit-learn library (Pedregosa et al., 2011). As it takes into account all metrics of each mouse, the LDA classifier is an effective tool for identifying multidimensional axes (Eigenvectors) that best separate experimental groups.

### Code and data availability

The software packages or algorithms used in this study are descripted in each corresponding section and freely available online. The Python codes and example data are available in a GitHub repository (see Extended Data 1).

### Statistical analysis

Data analysis was performed using JMP Pro 16 (SAS Institute). Data are expressed as means ± standard deviation. Behavioral responses between different odor conditions were compared using a one-way ANOVA or Student’s *t* test; *p *<* *0.05 was regarded as a statistically significant difference.

## Results

### Optimizing and evaluating the RF classifier

**Figure 3.**
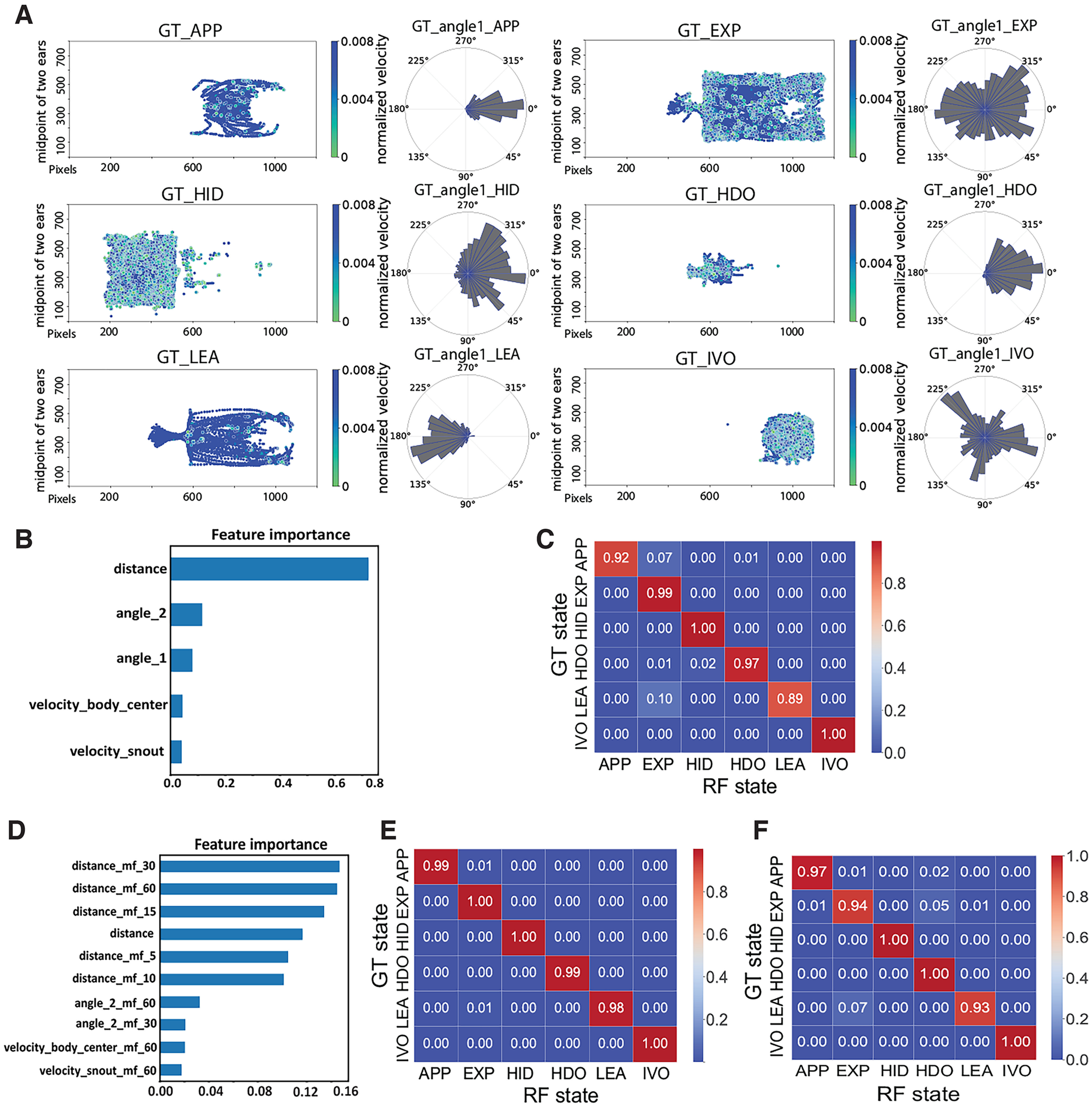
Plots of ground truth (GT) and performance of random forest (RF). ***A***, Graphical representations of the GT behavioral states. Each dot denotes the mouse position (midpoint between the ears) during manually annotated frames, with color indicating the normalized instantaneous velocity of the animal center. At right are polar plots indicating the direction of the mouse head relative to the horizontal axis. ***B***, Feature importance for the RF. ***C***, Confusion matrix for the RF versus the GT. ***D***, Feature importance of the optimized RF, which included rolling median filters of each derivative feature with temporal sliding windows (5, 10, 15, 30, and 60 frames) ***E***, Confusion matrix for the optimized RF versus GT. ***F***, Confusion matrix for the optimized RF versus GT for frames not included in the training set (*n* = 2218).

We manually annotated 12 top-down videos of mouse behavior (149,292 frames) in our behavioral arena to serve as a “ground truth” (GT) dataset. Tracking data from DeepLabCut were distilled to five derivative features, including the distance from the snout to the Petri dish, head angle relative to the sample Petri dish center, head angle relative to the horizontal image axis, and the snout and body center velocities (see Materials and Methods; Fig. 1*C*). In this scenario, the implementation of five behavioral features instead of raw positional information (*x/y* coordinates) resulted in a decrease in the computational cost of the ML model while also mitigating the impact of a moving Petri dish on the relative distance between the mouse body and Petri dish. Behavioral features of the GT were plotted for frames annotated as belonging to six behavioral states of a custom ethogram for the experimental setup (APP, HDO, EXP, HID, IVO, and LEA; see Materials and Methods; Fig. 3*A*).

In this study, we first tested the performances of several supervised ML models in classifying mouse risk assessment behavior using another training dataset (data not shown). The results showed that the RF model (0.9802) outperformed other models, such as K-nearest neighbors (0.9595), Support vector machine (0.9587), and Logistic Regression (0.9067). RF models have been widely applied in the classification of animal behavior in various testing contexts. (Valletta et al., 2017; Wang, 2019). We first evaluated the performance of the RF against GT data. We used the five derived features described above to train an RF classifier, achieving an overall accuracy of 0.9513. Feature importance analysis revealed that the distance between the snout and the sample Petri dish was the most informative feature for the RF model (Fig. 3*B*). Despite the high overall accuracy of the RF, the accuracy for each behavioral state was variable, ranging from 0.89 to 1.00 (Fig. 3*C*). Approximately 10% of “leaving” (LEA) and 7% of “approach” (APP) were misclassified as “exploration” (EXP). These misclassifications may have been a result of fewer training images for these states compared with others, as both LEA and APP were transient states occupying many fewer overall video frames compared with “hiding” (HID) and EXP. Another potential cause might be the inherent limitations of RF models, specifically their blindness to temporal features of the dataset. RF models distinguish class boundaries independently of neighboring data (i.e., timepoints before and after the analyzed frame), leading to a lack of temporal relations between outcomes (Rubinstein and Hastie, 1997; Lester et al., 2005). In an attempt to provide the model with some temporal information, we temporally smoothed the data with rolling median filters of variable window sizes (James et al., 2016; Cook et al., 2019; Fig. 3*D*). After inclusion of temporally filtered features, distances between the snout and Petri dish remained the most informative of the RF classification (Fig. 3*D*). This also improved the overall accuracy to 0.9931. Remarkably, the classification accuracy for APP and LEA reached 0.99 and 0.98, respectively (Fig. 3*E*). Furthermore, we tested this upgraded RF model to classify a small number of video frames that were novel to the RF model. The overall accuracy was 0.9970 (Fig. 3*F*). These results show that RF models achieve high classification accuracy in these conditions, and that RF models benefit from the inclusion of temporally smoothed data.

### Optimizing and evaluating the HMM

**Figure 4.**
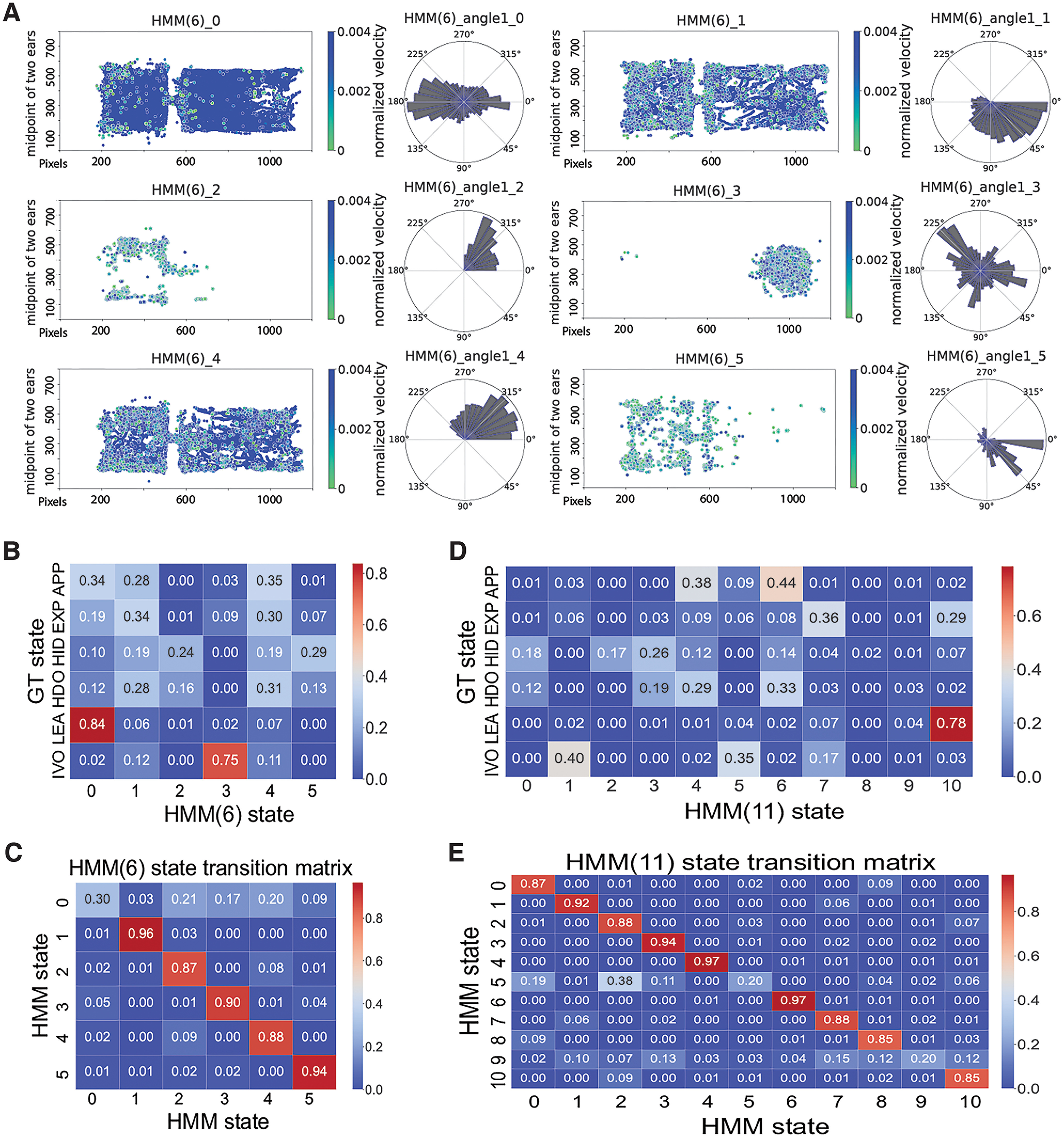
Performance of the hidden Markov model (HMM). ***A***, Graphical representations of HMM behavioral states for a 6-state classification [HMM(6)]. Each dot denotes the mouse position (midpoint between the ears) during manually annotated frames, with color indicating the normalized instantaneous velocity of the animal center. At right are polar plots indicating the direction of the mouse head relative to the horizontal axis. ***B***, Confusion matrix for the HMM(6) versus the GT. ***C***, State transition matrix of hidden Markov model for 6-state classification. ***D***, Confusion matrix for the HMM for 11-state classification [HMM(11)] versus the GT. ***E*,** State transition matrix of hidden Markov model for 11-state classification. Additional data can be found in Extended Data Figures 4-1, 4-2, and 4-3.

Although the RF model achieved high classification accuracy, its inherent limitations related to temporal components of behavior were of some concern (Sok et al., 2018). Hidden Markov models (HMMs) are another popular ML method for assessing highly dynamic behavioral data (Kim et al., 2010; P. Liu et al., 2016). HMMs infer transitions between hidden (latent) states that best predict observed data, potentially compensating for drawbacks of RF models. As an initial test of utility, we compared the performance between two ML models with/without leveraging temporal information (Fig. 4). The same five derivative features used for the RF models shown in Figure 3 were used to train HMMs (Fig. 4*B*,*C*). When given an equal number of latent states to the RF (6), the HMM identified some states that roughly matched GT states [especially “investigating-odor” (IVO) and “leaving” (LEA); Fig. 3*B*]. However, most HMM states were not clearly matched to a hypothesis-related state (Fig. 4*A*), which was reflected in the confusion matrix between the 6-state HMM and the 6-state RF classification RF (Fig. 4*B*). For the HMM state transition, most of HMM states, except for state 0, exhibited high probability of self-transition (Fig. 4*C*). Given the capacity for HMMs to identify hypothesis-related states outside our RF state definitions, and because the number of latent states is defined by users, we generated HMMs with increasing numbers of hidden states in two batches, ranging from 6 to 12 states (Extended Data Fig. 4-1*A*,*B*) and 13 to 24 states (Extended Data Fig. 4-3*A*,*B*). Despite the additional flexibility, these HMMs did not clearly identify new or missing hypothesis-related states, which was reflected in the confusion matrices comparing the HMM output to six-state RF output. (Fig. 4*D*,*E*; Extended Data Figs. 4-1*C*, 4-2*A*,*B*, 4-3*C*). Thus, despite advantages related to sequential information, HMM labels had limited capacity to support hypothesis testing in these conditions.

10.1523/ENEURO.0335-22.2022.f4-1 Extended Data Figure 4-1 Overview of hidden Markov model for 11-state classification. ***A***, Expectation-Maximization (EM) algorithm for hidden Markov model for 11-state classification [HMM(11)] relative to the number of training iterations. ***B***, Bayesian Information Criterion (BIC) scores of HMM(11) relative to the number of hidden states. ***C***, Graphical representations of hidden behavioral states, as predicted by HMM(11). For each behavioral state, dots represent the midpoint of two ears, and the color represents the velocity of the body center. The right polar plot represents the angle between the head direction vector and the horizontal *x*-axis. Download Figure 4-1, TIF file.

10.1523/ENEURO.0335-22.2022.f4-2 Extended Data Figure 4-2 Performance of the hidden Markov model for 23-state classification. ***A***, Confusion matrix for the HMM for 23-state classification [HMM(23)] versus the GT. ***B***, State transition matrix of hidden Markov model for 23-state classification. Download Figure 4-2, TIF file.

10.1523/ENEURO.0335-22.2022.f4-3 Extended Data Figure 4-3 Overview of hidden Markov model for 23-state classification. ***A***, Expectation-Maximization (EM) algorithm for hidden Markov model for 23-state classification [HMM(23)] relative to the number of training iterations. ***B***, Bayesian Information Criterion (BIC) scores of HMM(23) relative to the number of hidden states. ***C***, Graphical representations of hidden behavioral states, as predicted by HMM(23). The dots represent the midpoint of two ears, and the color represents the velocity of the body center. The right polar plot represents the angle between the head direction vector and the horizontal *x*-axis. Download Figure 4-3, TIF file.

### Hybrid modes reHMM and reHMM+

**Figure 5.**
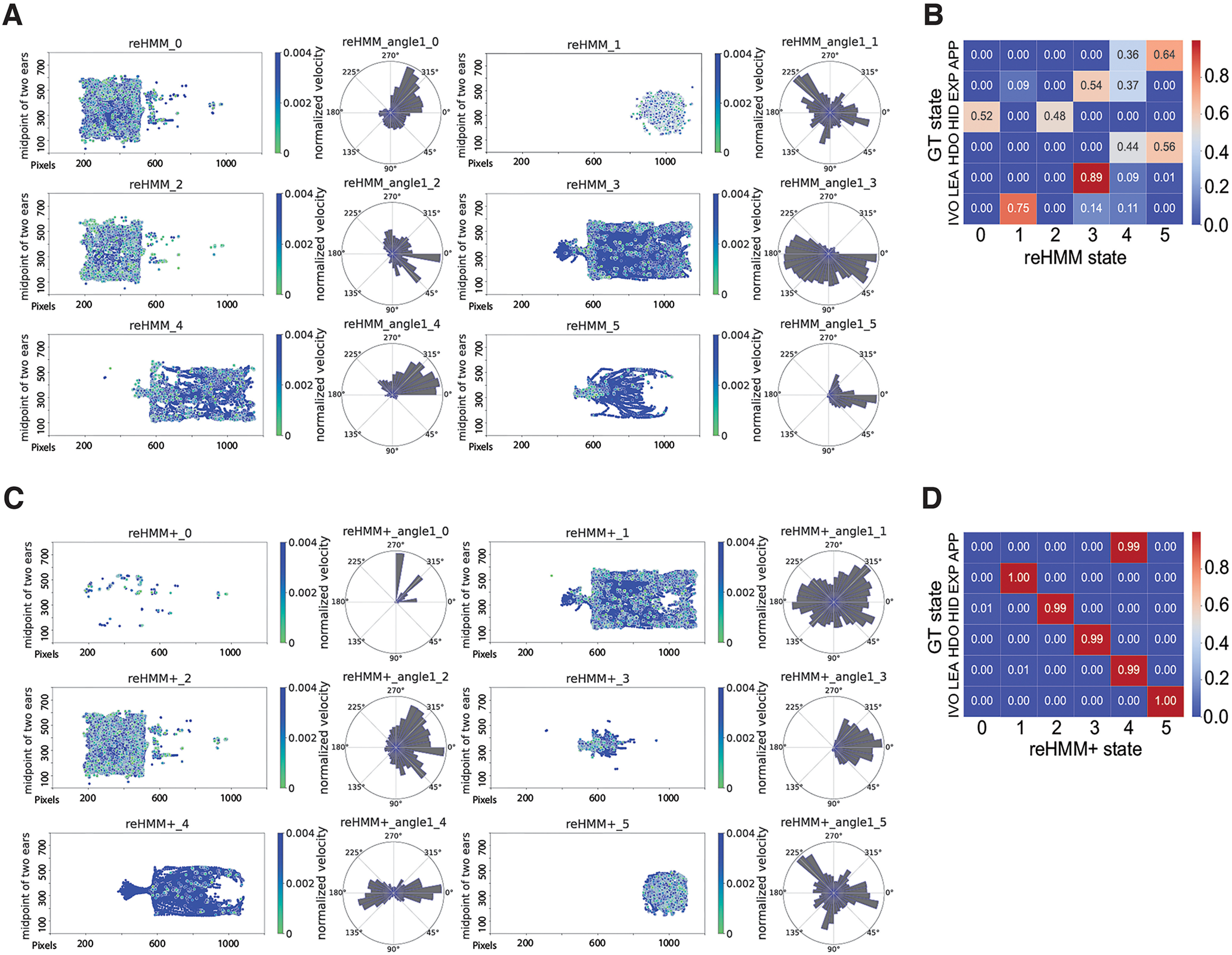
Performance of reHMM and reHMM+. ***A***, Graphical representations of hidden behavioral states, as predicted by the reHMM. Dots represent the midpoint of the two ears and the color represents the velocity of the body center. Polar plots represent the angle between the head direction vector and the horizontal *x*-axis. ***B***, Confusion matrix for reHMM versus the GT. ***C***, Graphical representations of hidden behavioral states, as predicted by the reHMM+. ***D***, Confusion matrix for reHMM+ versus the GT.

Given the limitations of RF and HMM strategies, we next considered a hybrid ML models, which have been demonstrated to outperform either alone (Lester et al., 2005; Antos et al., 2014; Sok et al., 2018). We first used the outputs of RF and HMM to train a new HMM model for 6-state classification, named “reHMM” (Fig. 5). reHMM model output showed a closer match between reHMM states and GT compared with HMM-alone (Fig. 5*A*,*B*). For example, the reHMM states 0 and 2 together split frames associated with the GT HID state, accounting for 52% and 48%, respectively (Fig. 5*B*). The reHMM state 5 included 64% of APP and 56% of HDO, which share the feature that the mouse body is oriented toward odorant cues.

Given the improvement of reHMM compared with the 6-state HMM alone, we sought to further enhance the reHMM model’s interpretability. Leontjeva and Kuzovkin reported that combining dynamic and static features enables classification models to simultaneously capture static information and temporal dynamics (Leontjeva and Kuzovkin, 2016). In our effort to optimize the RF, the distance between the mouse snout and the stimulus order was the most informative static feature, so we added this feature to the RF-only and HMM-only predictions (creating a three-dimensional matrix). This input matrix, including the predictions of RF-only, HMM-only, and the distance measurement, was used to train a reHMM variant, named “reHMM+” (Fig. 5*C*,*D*). reHMM+ states were highly interpretable and mapped strongly to GT states, suggesting similar classifying strength of the reHMM+ to the RF model (Fig. 5*C*,*D*). For example, the reHMM+ state four contained both APP and LEA, which could be regarded as a back-and-forth state. IVO state was exclusively distributed in the reHMM+ state 5, providing an avenue to decipher IVO patterns and transition frequencies between IVO and others. Overall, this layered, hybrid reHMM+ model showed the capacity to classify behaviors in these experimental conditions with high interpretability and accuracy, incorporating static and temporal features to achieve its predictions.

### TMT induced a heightened level of fear-like behavior in mice

**Figure 6.**
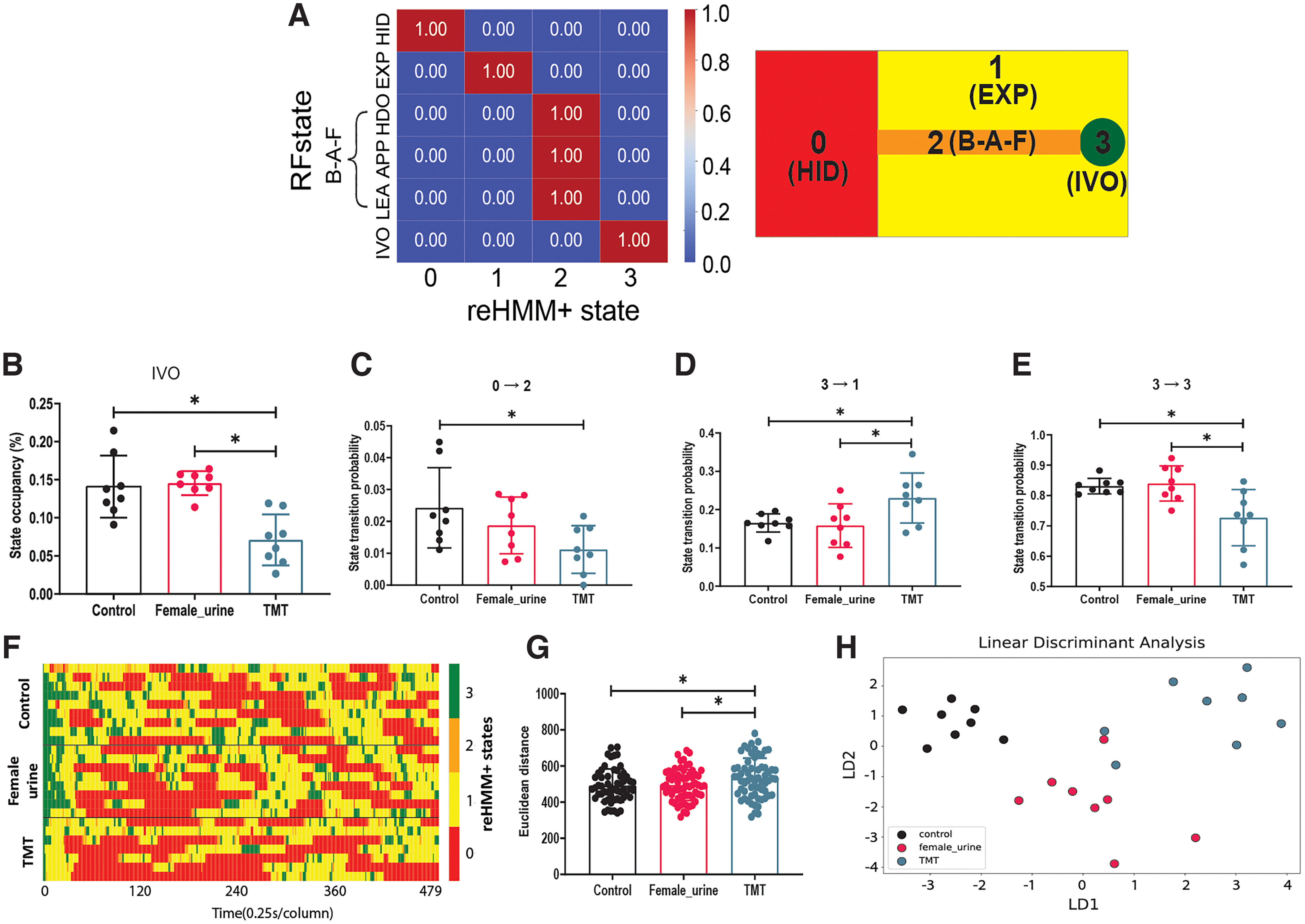
TMT induced a heightened level of fear-like behavior in mice. Analysis was conducted for a 2-min window starting with the first IVO event. ***A***, Confusion matrix for the merged reHMM+ states versus the RF states. For simplicity, the three reHMM+ states matching the RF states approaching (APP), heading-out (HDO), and leaving (LEA) were merged into the back-and-forth (B-A-F) state. Right, Graphical representation of the interpretation of merged reHMM+ states. Red indicates state 0 [hiding(HID)]. Yellow indicates state 1 [exploration(EXP)]. Orange indicates state 2 [back-and-forth(B-A-F)]. Green indicates state 3 [investigating-odor (IVO)]. ***B***, Occupancy analysis for the reHMM+ state IVO. ***C–E***, Transition probabilities between listed reHMM+ states. ***F***, Heatmap illustrating behavioral sequence aligned to the first IVO event. Each row indicates one mouse. Each column indicates the time (0.25 s). The color code is identical to the description in panel ***A***. ***G***, Behavioral sequence similarity, as evaluated by Euclidean distance. ***H***, Plot of linear discriminant analysis; *p <* *0.05, one-way ANOVA followed by multiple comparisons tests. For this experiment, 24 mice (*n* = 8 for each treatment group) were available for analysis. Additional data can be found in Extended Data Figure 6-1.

We next sought to test the reHMM+ model to evaluate its performance in mouse risk assessment behavioral assays (Fig. 6). To simplify interpretation, the reHMM+ states representing HDO, APP, and LEA were merged into one state reHMM+ state 2. This state included transition states between the entry to the safe area and the odor object, essentially a back-and-forth (B-A-F) state. States 0, 1, and 3 effectively encapsulated HID, EXP, and IVO, respectively. For visualization, each reorganized reHMM+ state was also assigned a color code; red represented avoidant HID and yellow represented neutral EXP, respectively. Orange represented B-A-F state and green represented attractive IVO, respectively (Fig. 6*A*).

10.1523/ENEURO.0335-22.2022.f6-1 Extended Data Figure 6-1 Additional analysis of male mouse behavioral responses to TMT and female mouse urine. ***A***, Interval between two consecutive IVOs. ***B***, Duration of each IVO. ***C***, Total number of IVO. ***D***, Latency of the first IVO. ***E***, Total movement distance; *p <* *0.05, one-way ANOVA followed by multiple comparison’s tests. A total of 24 mice (*n* = 8 for each treatment group) were available for analysis. Additional data can be found in Extended Data Figure 6-1 and Extended Data 6-2. Download Figure 6-1, TIF file.

In an initial proof-of-concept experiment, TMT, female mouse urine, and pure water were used as test odorants. TMT, a compound derived from red fox feces, has been widely used to induce fear-like behavioral responses in rodents, such as freezing, avoidance, and defensive burying (Fendt et al., 2005). Consistent with previous studies, the reHMM+ output showed that mice spend less time on investigating TMT compared with female urine and pure water (Fig. 6*B*). Moreover, reHMM+ revealed that mice had a lower probability of transiting from state 0 to 2 and state 3 to 3, while a higher probability of switching from state 3 to 1 in the presence of TMT (Fig. 6*C–E*; Extended Data Fig. 6–2). These observations suggest that TMT-exposed mice were inclined to stay in HID and leave IVO. During the first 2 min following the first IVO event (the first close investigation of the test odorant), the average intervals between two consecutive IVOs were larger in TMT-treated mice compared with pure water (Extended Data Fig. 6-1*A*). Also, the average duration of IVO was much shorter for mice encountering TMT compared with animals faced with water and female urine (Extended Data Fig. 6-1*B*). These data indicate that mice displayed less frequent and shorter investigative responses to TMT. On the other hand, we did not find differences in the total number of IVO events, the latency to the first IVO event, or total movement distance between the three treatments (Extended Data Fig. 6-1*C–E*). We next measured the Euclidean distance between the first-IVO-aligned reHMM+ classifications to investigate the behavioral sequence similarity in each odor treatment, finding that the behavioral sequence of TMT-treated mice differed from those of mice treated with pure water and female urine (Fig. 6*F*,*G*). By applying this analytical workflow, many metrics were produced, including reHMM+ state occupancy, the transition probability of reHMM+ states, IVO frequency, IVO duration, IVO latency, the total number of IVO, total movement distance, and behavioral sequence similarity. Thus, we used the LDA classifier to compare the overall behavioral difference across all behavioral metrics. LDA analysis revealed that the behavioral outputs from mice confronted with different odor cues could be readily distinguished (Fig. 6*H*). These data show that the reHMM+ analytic workflow is well-suited for behavioral pattern analysis in response to appetitive and aversive odorants.

### Snake feces stimulated risk assessment behaviors in mice

**Figure 7.**
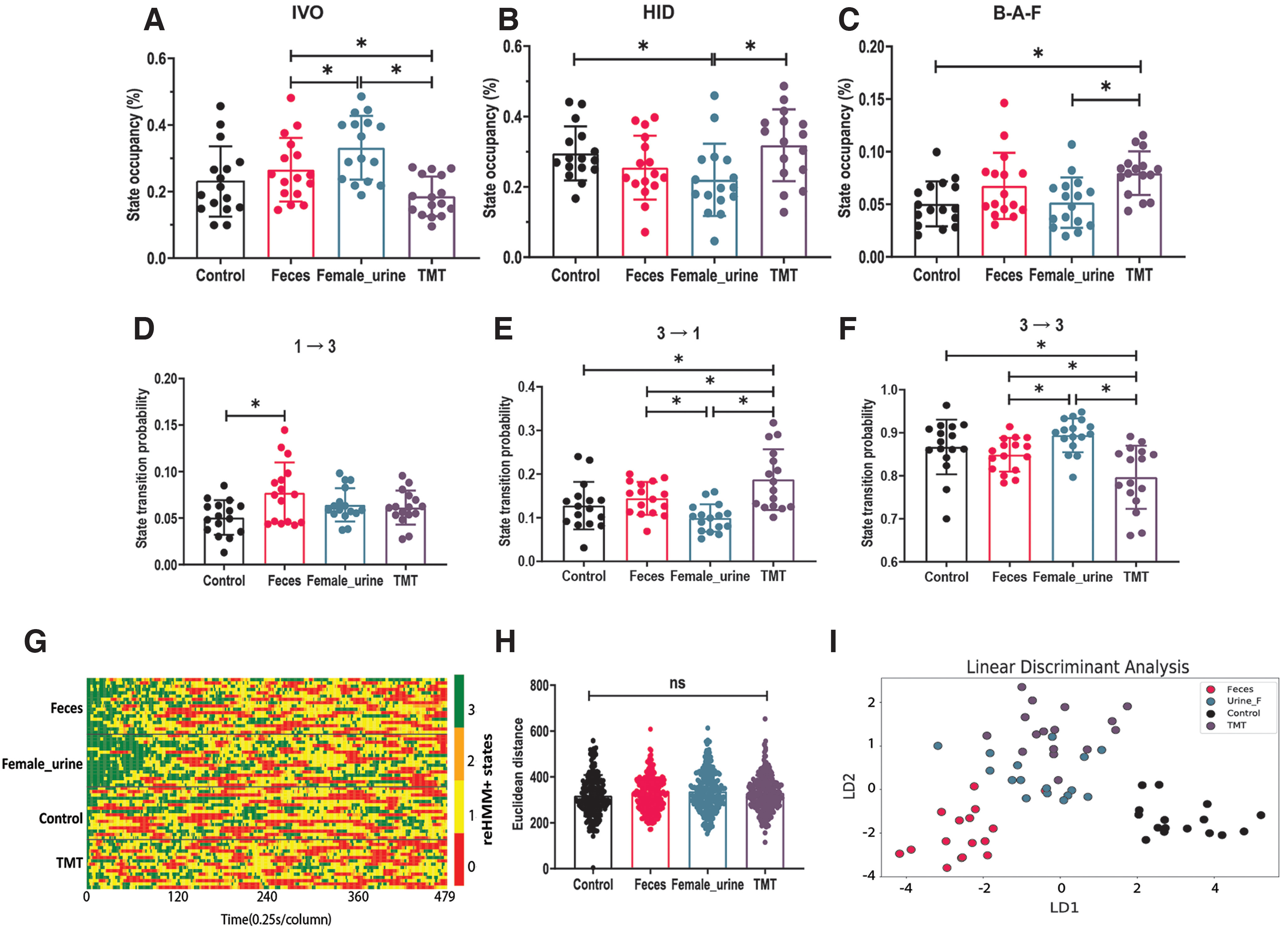
Snake feces stimulated risk assessment behaviors in mice. ***A–C***, Occupancy analysis for listed reHMM+ states. ***D–F***, Transition probabilities between listed reHMM+ states. ***G***, Heatmap illustrating behavioral sequences aligned to the first IVO event. Each row indicates one mouse. Each column indicates the time (0.25 s). Red indicates state 0 [hiding(HID)]. Yellow indicates state 1 [exploration(EXP)]. Orange indicates state 2 [back-and-forth(B-A-F)]. Green indicates state 3 [investigating-odor (IVO)]. ***H***, Behavioral sequence similarity, as evaluated by Euclidean distance. ***I***, Plot of linear discriminant analysis; *p <* *0.05, one-way ANOVA followed by multiple comparisons tests; ns: not significant. For this experiment, 16 mice were available for analysis. Additional data can be found in Extended Data Figures 7-1 and 7-2.

Quantifying threat assessment behavior can be a challenging task, especially in the context of complex chemosignal blends that laboratory strains of mice have never encountered in their natural context (e.g., predator cues). We investigated the mouse behavioral responses to snake feces, a predatory odor cue. In this experiment, TMT, female mouse urine, and pure water were used as negative, positive, and neutral controls, respectively. In this experiment, each animal was exposed to all 4 odorants in a pseudorandom order (the exception was that negative control was always first and TMT always last). reHMM+ state occupancy analysis revealed that snake feces-treated mice spent more time in IVO state than TMT, whereas less time compared with female urine (Fig. 7*A*). Similar to the observations of the first pilot study, exposure to TMT was associated with a higher occupancy of B-A-F, but a lower level of occupancy in IVO, compared with female urine (Fig. 7*A–C*). Mice investigating female mouse urine displayed higher occupancy of IVO while lower HID relative to pure water (Fig. 7*A*,*B*). reHMM+ demonstrated that snake feces caused a higher probability of transiting from state 1 to 3 compared with pure water (Fig. 7*D*; Extended Data Fig. 7-2). Compared with TMT, snake feces-encountered mice displayed a lower probability of leaving state 3 (3 to 1; Fig. 7*E*). Interestingly, the probability of staying in state 3 (aligned with the “investigating-odor” RF condition) in response to snake feces was higher than TMT, but lower than female urine (Fig. 7*F*).

Also, this analysis suggested that snake feces caused a higher frequency of IVO (shorter IVO interval) and more total number of IVO relative to pure water (Extended Data Fig. 7-1*A–C*). This suggested that the animals were actively assessing the snake feces, and that they found it mildly threatening compared with conspecific cues. In these conditions, TMT treatment was associated with increased overall IVO frequency, but an overall decreased IVO duration compared with pure water (Extended Data Fig. 7-1*A–D*). This suggested that in these conditions, where mice are habituated to their environment by a series of control and odorant presentations, TMT is not as overtly fear-inducing as it is in other conditions. As expected, female mouse urine caused the longest IVO duration among all treatments (Extended Data Fig. 7-1*B*). In these conditions, we observed no difference in the total movement, suggesting that animals did not freeze for long periods of time in these conditions, even in the presence of the well-established aversive TMT odorant (Extended Data Fig. 7-1*E*).

10.1523/ENEURO.0335-22.2022.f7-1 Extended Data Figure 7-1 Additional analysis of male mice behavioral responses to female mouse urine, snake feces, and TMT. ***A***, Interval between two consecutive IVOs. ***B***, Duration of each IVO. ***C***, Total number of IVO. ***D***, Latency of the first IVO. ***E***, Total movement distance; *p <* *0.05, one-way ANOVA followed by multiple comparison’s tests. For this experiment, 16 mice were available for analysis. Download Figure 7-1, TIF file.

First-IVO-aligned behavioral sequence analysis indicated that the behavioral responses to all test and control odorants were not generally distinguishable from each other (Fig. 7*H*,*I*). LDA analysis, on the other hand, which incorporated a broader range of metrics, revealed that behavioral responses to snake feces were separable from other odorants, including TMT (Fig. 7*J*). These studies show that mice exposed to multiple odorant presentations in this threat assessment assay respond differently to odorants than when they encounter them naively. The data also suggest that mice respond to novel predatory cues with an increased behavioral trend of risk assessment, not overt aversion.

### Snake feces extract promoted risk assessment behaviors in mice

**Figure 8.**
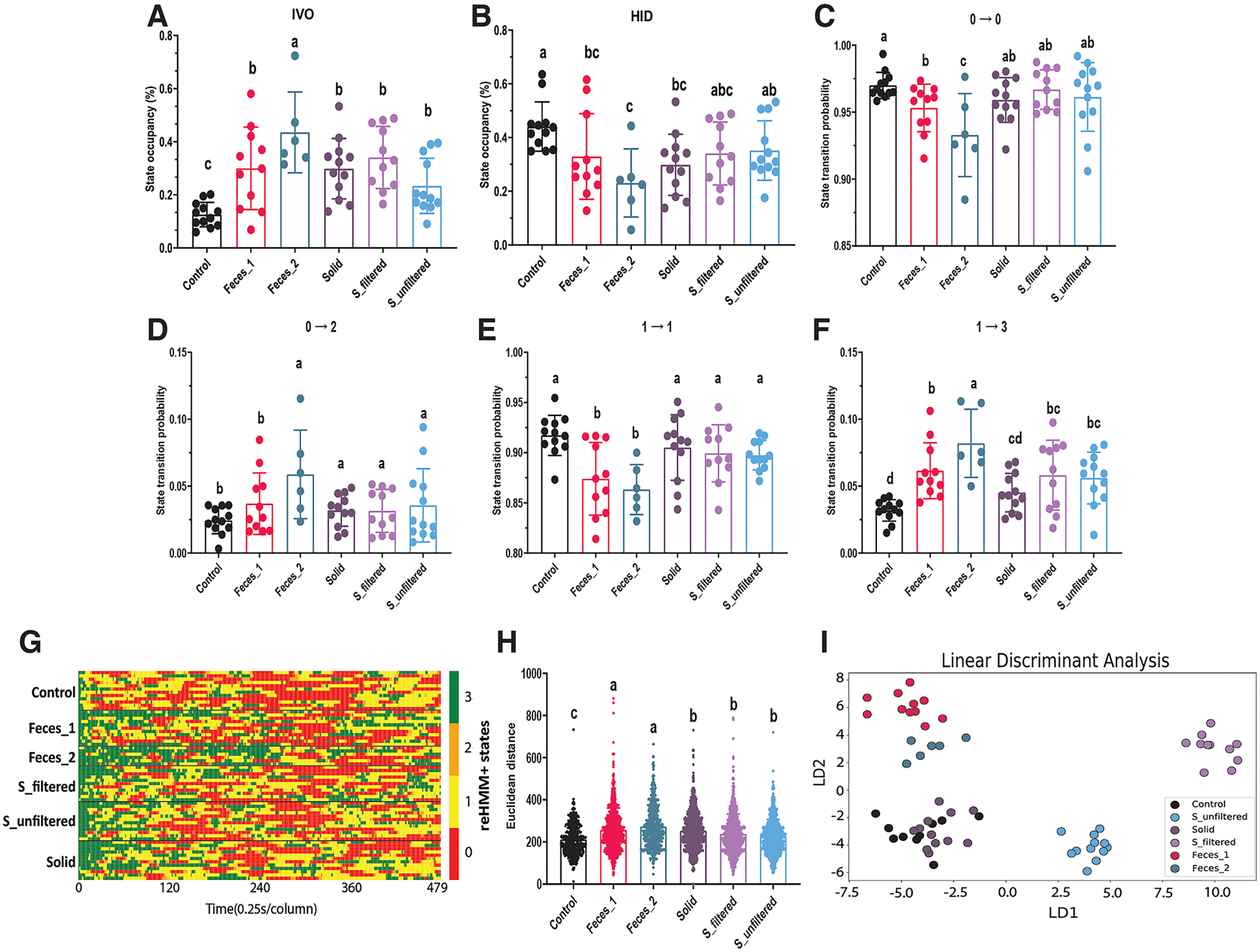
Snake feces extract promoted risk assessment behaviors in mice. ***A***, ***B***, Occupancy analysis for listed reHMM+ states. ***C–F***, Transition probabilities between listed reHMM+ states. ***G***, Heatmap illustrating behavioral sequence. Each row indicates one mouse. Each column indicates the time (0.25 s). Red indicates state 0 [hiding(HID)]. Yellow indicates state 1 [exploration(EXP)]. Orange indicates state 2 [back-and-forth(B-A-F)]. Green indicates state 3 [investigating-odor (IVO)]. ***H***, Behavioral sequence similarity, as evaluated by Euclidean distance. ***I***, Plot of linear discriminant analysis; Letter codes (e.g., “a”, “b”, “ab”, etc.) identify statistically distinct groups as assessed by one-way ANOVA followed by multiple comparisions tests. For this experiment, six mice were available for analysis. Additional data can be found in Extended Data Figures 8-1 and 8-2.

A major goal in chemosensory neuroscience is to identify specific chemosignals that drive behavioral changes. To support the eventual identification of novel predatory chemosignals, we introduced animals to fractions of snake feces, including unfiltered and sterile filtered extracts, as well as residual solids (Fig. 8). Snake feces and pure water were used as positive and negative controls, respectively. A second snake fecal treatment from a separate species was used in place of TMT as the final positive control stimulus. Model outputs showed that filtered and unfiltered extracts and the remaining solid caused higher IVO occupancy than pure water, but less time spent in IVO compared with snake feces (Fig. 8*A*). Both snake feces treatments were associated with a lower HID occupancy and probability of staying in state 0, compared with pure water (Fig. 8*B*,*C*; Extended Data Fig. 8-2). Filtered and unfiltered extracts induced a higher probability of transiting from state 1 to 3 and from state 0 to 2, compared with pure water (Fig. 8*D*,*E*), whereas mice treated with filtered and unfiltered extracts displayed a higher probability of staying in state 1 relative to two snake feces (Fig. 8*F*). Similar to the study shown in Figure 6, snake feces increased the total number of IVO events compared with pure water (Extended Data Fig. 8-1*B*,*C*). Unfiltered extracts also increased IVO events and IVO duration, while filtered extracts only increased the total number of IVO (Extended Data Fig. 8-1*B*,*C*). No difference in IVO interval, the latency of IVO and movement distance was observed (Extended Data Fig. 8-1*A*,*D*,*E*). The first-IVO-aligned behavioral sequences of filtered and unfiltered extracts and the remaining solid differed from pure water and two snake feces (Fig. 8*G*,*H*). Finally, LDA revealed that the behavioral outputs of all odorants could be distinguished, with the exception of the remaining solid and pure water control conditions (Fig. 8*I*). In all, these results suggest filtered and unfiltered extracts induced quantitatively different risk assessment behaviors than feces, suggesting that behavioral responses to complex odorant mixtures depend on the specific chemosignals present, not the presence or absence of a single component of the mixture.

10.1523/ENEURO.0335-22.2022.f8-1 Extended Data Figure 8-1 Additional analysis of male mouse behavioral responses to snake feces and its extracts. ***A***, Interval between two consecutive IVOs. ***B***, Duration of each IVO. ***C***, Total number of IVO. ***D***, Latency of the first IVO. ***E***, Total movement distance; *p <* *0.05, one-way ANOVA followed by multiple comparison’s tests. For this experiment, six mice were available for analysis. Download Figure 8-1, TIF file.

## Discussion

Recent advances in ML have tremendously facilitated our ability to measure and understand animal behavior (Wang, 2019). Multiple ML-based software packages, such as DeepLabCut (Mathis et al., 2018), Social LEAP (SLEAP; Pereira et al., 2022), and DeepPoseKit (Graving et al., 2019), can efficiently and accurately extract animal movement and posture information from videos. Despite this progress, there are still challenges for analyzing and interpreting complex, diverse, and high-dimensional behavior datasets (Pérez-Escudero et al., 2014; Nakamura et al., 2016). Currently, there are several analytical software packages available that use either unsupervised [e.g., B-SOiD (Hsu and Yttri, 2021), MoSeq (Wiltschko et al., 2020)] or supervised [e.g., DeepEthogram (Bohnslav et al., 2021), SimBA (Nilsson et al., 2020)] ML for pose estimation and classification of behavioral data. In this study, a novel layered, hybrid analytic workflow was developed with the goal of incorporating the advantages of supervised and unsupervised ML models to improve the accuracy and interpretability of animal behavior quantification.

In general, the objective of a study and the format of behavioral data determine the best analytic strategies and tools. In many cases, experimenters carefully design conditions that they hypothesize will generate changes in predefined behavioral states of interest. In these cases, supervised classifiers, such as RF, are generally well-matched to the overall goal (Nilsson et al., 2020; Winters et al., 2022). In our study, a well-trained RF achieved relatively high initial accuracy (>95%), but had high error rates in highly dynamic/transient states (e.g., LEA and APP). Because RF classifiers do not integrate temporal/sequential components in their predictions, we applied a series of rolling median filters to input data to create temporally smoothed copies of the animal’s body positions and orientations (i.e., some temporal information). This boosted the classification performance (to 0.9931) and improved prediction accuracy for transient states (e.g., LEA and APP). Since animal movement is highly dynamic, and often includes fast-switching between behavioral states (Fauchald and Tveraa, 2006), improving the accuracy of supervised models in classifying transient states has great value.

In experiments where the experimental goal is to more generally explore the structure of animal behavior, unsupervised models, such as HMMs, are well suited (Wiltschko et al., 2020; Findley et al., 2021). The main caveat of HMMs is that the outcomes may not map directly to specific behaviors of interest. In this study, our experimental design was specifically intended to assess mouse threat assessment, but HMM states mapped poorly onto our user-defined ethogram (GT states; Fig. 4). Increasing the number of HMM states, in hopes of finding some latent states with high interpretability, did not succeed (Fig. 4). This experience was the primary motivation for pursuing a hybrid model. We found that the reHMM model improved interpretability, even with a small number of states (Fig. 5*A*,*B*). reHMM+, which incorporated RF, HMM, and a single high-importance input feature, resulted in excellent interpretability, nearly matching user-defined GT states (Fig. 5*C*,*D*). The high degree of interpretability of reHMM+ comes with the added benefit of being backed by a dynamic model. The layered architecture reduced burdens (computational time and end-user evaluation) associated with determining the best parameters for HMM training and application (e.g., the number of hidden states). Furthermore, by taking advantage of high-value but low-computational cost components (RF output, high-importance raw features), reHMM+ has substantially reduced complexity and computational cost, making it highly flexible and adaptive.

Our behavioral tests demonstrated that reHMM+ is well-suited for behavioral pattern analysis in mouse risk assessment behavior. TMT-induced behavioral responses in these conditions ran counter to typical findings, specifically that TMT causes hiding/freezing and total odor avoidance (Morrow et al., 2000). In our conditions, TMT-exposed mice tended to stay away from the odorant, but adopted a rapid-and-short investigative pattern to sample (IVO) TMT (Fig. 6). Using reHMM+, we also find that mice react to potentially risky cues (snake feces and its extracts) with a different pattern of sampling and exploring than TMT, consistent with risk assessment (Fig. 7). A major benefit of reHMM+ in this context is that traditional measurements from user-defined ethograms (time spent in state, number of times entering state) can be evaluated along with state transition information, producing a broad and deep behavioral profile. Combined, the data generated by the reHMM+ workflow are capable of distinguishing responses to odors with similar overall valence but variably overlapping molecular components (e.g., TMT vs snake feces, etc.). In the future, we anticipate that this analytical approach will allow identification of novel behavioral effects of chemosensory secretions, and ultimately the brain processes that underlie diverse chemosignal-mediated behaviors.

The benefits of reHMM and reHMM+ workflows make them attractive in experiments designed to investigate specific behavioral hypotheses, but they also have several noteworthy limitations. First of all, we developed reHMM+ using specific ML models with complementary strengths and weaknesses (e.g., HMM and RF). Although reHMM and reHMM+ models do not require extensive tuning of model hyperparameters, their performance still varies depending on several factors, such as feature selection and the specific behavioral ethogram chosen. Additionally, the choice of the tracking feature to add into the reHMM+ layer may qualitatively affect the end result. Here, the reHMM+ model incorporates the most important feature in RF classification, the distance from the mouse nose to the sample dish. This choice resulted in HMM states that closely matched the GT (ethogram) states, improving the mating-rate to GT states. This had the consequence of seemingly reducing the influence of the first-order HMM states (Figs. 5, 6). Meanwhile, in the the scenario of combinatorial explosion of behavioral features, it would result in difficulty to select an ideal feature or feature combination for reHMM+ model. One of solution to address this potential issue is to incorporate the Explainable Artificial Intelligence (XAI) technique, such as SHapley Additive exPlanations (SHAP), to explore more detailed and explainable information about feature importance, thereby assisting feature selection (Goodwin et al., 2022). Third, since a major benefit of HMMs is objective assignment of latent states that may have undiscovered important neurobiological underpinnings (Leos‐Barajas et al., 2017), the reHMM+ output may not always be superior to reHMM. The value of the reHMM+ approach depends on the degree to which investigators wish to adhere to subjective, but interpretable, behavioral states.

Overall, we found that applying layered, hybrid ML workflows in our experimental context unveiled distinctive mouse behavioral patterns induced by established and experimental chemosensory stimuli, indicating diversity in the way chemical signals modulate mouse risk assessment behavior. Because of their inclusion of user-specified ethograms, we anticipate that the reHMM and reHMM+ models will be especially powerful in the context of more complicated experimental settings, such as multianimal social interaction tests. Ultimately, we find that layered, hybrid analytic workflows improve the depth and reliability of ML models and expand the ability to explore the dynamic architecture of animal behavior.

10.1523/ENEURO.0335-22.2022.f6-2 Extended Data 6-2 This is a .csv file containing the ReHMM+ transition matrix presented in Figure 6. Download Extended Data 6, CSV file.

10.1523/ENEURO.0335-22.2022.f7-2 Extended Data 7-2 This is a .csv file containing the ReHMM+ transition matrix presented in Figure 7. Download Extended Data 7, CSV file.

10.1523/ENEURO.0335-22.2022.f8-2 Extended Data 8-2 This is a .csv file containing the ReHMM+ transition matrix presented in Figure 8. Download Extended Data 8, ZIP file.

10.1523/ENEURO.0335-22.2022.ed1 Extended Data 1 This .zip directory contains six files with example data and code used in this manuscript, along with selected outputs from the reHMM+ workflow. The file named DeepLabCut_raw_data.csv contains a spreadsheet with consolidated raw tracking data outputs from DeepLabCut, which were the inputs for the results presented in Figures 3 and 4 and Extended Data Figures 4-1, 4-2, and 4-3. The files Analytic workflow_1_define behavioral feature.ipynb, Analytic workflow_2_RF and HMM classification.ipynb, and Analytic workflow_3_reHMM and reHMM+ classification.ipynb contain Python notebooks implementing the workflows presented in Figures 3–8 and associated Extended Data Figures Line 43 in Analytic workflow_1_define behavioral feature.ipynb is a generic call to load data, which can be used to load DeepLabCut_raw_data.csv. Executing this code will generate a series of .csv file outputs, which are used later. Line 41 in Analytic workflow_2_RF and HMM classification.ipynb is a generic call to load the .csv file outputs from Analytic workflow_1_define behavioral feature.ipynb. Executing this code will generate a series of .csv file outputs, which are the outputs of the initial RF and HMM models. This also generates .sav files, which contain the model specifications themselves for future use if needed. Line 76 of Analytic workflow_3_reHMM and reHMM+ classification.ipynb is a generic call to load the .csv file outputs from Analytic workflow_2_RF and HMM classification.ipynb. Executing this code will generate .csv files, which contain the reHMM and reHMM+ outputs, as well as .sav files which contain the model specifications themselves for future use if needed. Plotting and analysis of results is user specific, and can be done as needed using general plotting and statistical analysis functions available in Python (e.g., numpy, matplotlib, etc.). Download Extended Data 1, CSV file.

## Synthesis

Reviewing Editor: David Schoppik, New York University Decisions are customarily a result of the Reviewing Editor and the peer reviewers coming together and discussing their recommendations until a consensus is reached. When revisions are invited, a fact-based synthesis statement explaining their decision and outlining what is needed to prepare a revision will be listed below. The following reviewer(s) agreed to reveal their identity: NONE.

The reviewers both felt that the manuscript merits publication and had a number of comments and suggestions for ways to strengthen the work. Please address them individually.

1. The reviewers both felt that the manuscript did not sufficiently make the advance clear, nor was it clear how scalable/generalizable the work was likely to be. Both reviewers felt that the manuscript would be stronger for comparison with other approaches. They had suggestions for specific analyses, as well as ways to broaden the Discussion.

(a) discussion of B-SOID or Deepethogram, or JAABA/GentleBoost algorithm (Branson lab)

(b) comparison of the results to GT beyond the initial training in novel videos? Specifically with respect to #2, the authors are encouraged to classify a small number of frames and check the accuracy. The reviewer felt that “comparison to GT data not in the training set is essential.”

(c) discussion of the appropriateness of the approach, and additional comparison to other HMM-based approaches. I’ll quote here from one of the reviewers who had a number of good suggestions:

“The problem that the authors are studying is not that hard of a ML problem and their RF itself does a really good job, particularly, after their temporal filtering (Figure 2E). The small improvements that they see are not necessarily game-changing. The classification of three states into a single state can be discovered in the Transition Probability matrix. Further, the HMM in the first step won’t be that helpful because HMM states are too short-lived. The authors can check this by plotting the duration in each state, which are likely to be very short. If true, comparison to hierarchical versions of HMM or HHMM like employed in Bhandawat et. al. would be important. Alternatively, there may be short-term structure in transition probabilities of the HMM that corresponds to each human defined 6 states - particularly in HMM with many states. You might observe that only some of the state transitions are present”

(d) consideration of how other labs have leveraged HMMs. Again, quoting from a reviewer: “I am sure the authors know this and may not have meant it but it seems that they make a big deal about how HMM states are uninterpretable. Comparing an unsupervised model to a supervised model in this manner is not appropriate. Also, the reason to use HMMs is to discover things in the data that are not that obvious to humans - like speed trends etc.”

2. There were three additional questions about Methods

As a human annotator, a starkly evident feature for many of the different states is the distance to the petri dish, followed by the animal’s orientation to it. Is it then surprising that the distance is a key feature (see Figure 2)? Another way of framing this is: in an arbitrary behavioral situation, what if we didn’t know what the key feature was in an intuitive way?

Why is one of the states called “sniffing odor”, when there is no information with respect to the animal sampling the odor? It would be more appropriate to call it “Interacting with or investigating odor”

How did the authors deal with occlusion of body parts, when for example, the animal is grooming? How did the authors deal with frames where points were not reliably tracked?

3. The reviewers had general comments on the figures:

The reviewers found the quantification & visualization lacking, particularly the bar plots in Figures 5-7. The authors are *strongly* encouraged to consider estimation statistics / visualization per e.g. “Moving beyond p-values: data analysis with estimation graphics” by Adam Claridge-Chang et. al. to provide a more accurate picture of their data.

4. As well as specific comments on the figures:

In Figure 2A, midpoint of the two ears are being plotted and it is not actually the y-axis. Correct? This was a little confusing. Marking the box as in Figure 1 would be helpful.

The data presented in Fig 5-7 are very detailed; it might be good to conclude with a summary figure, collecting the major findings from figs 5-7.

Figure 5-1: units in 0.25 second is not intuitive.

5. Finally, the reviewers had specific comments on the text:

The acronyms employed AP etc are not intuitive. Maybe three letter acronyms like the one authors use for Exploration would be easier to keep track of

More information on the LDA is necessary

Line 7 and throughout: Is RF really the best supervised model for classifying behaviors (well, it’s really for poses to be precise)? It’s not clear why the emphasis is so much on this one, and justifying it might be good.

Line 26 (and throughout): HMMs are not really classifiers - they are state models.

Lines 42/43: It will be fair to also cite LEAP by Pereira et al here (Nat Meth 16:117). The authors used the outputs of DLC, but in principle, these methods would work for other pose estimation methods such as LEAP. Also, Mathis et al is incorrectly double cited - please correct this and any other reference/citation issues.

Line 54: Siddiqui and Medioni looks more like a pose-estimation rather than behavior retrieval algorithm? Please clarify.

Lines 62/63: The authors make an important point about the differences in classifying behaviors based on frames without taking into account sequences, vs methods to do this taking into account temporal information. But pitching it as RF vs HMM seems odd - there are many other sorts of classifiers of poses based on single frames than RFs. Perhaps it will help the reader to get this point better by pointing out that RF is one of many classifiers based on single frames? Bottom line: it’s not really RF vs HMM, but static classifiers vs ones based on dynamics (ideally with different temporal scales).

Line 72: all of these applications are vast, so hard to cite specific ones - but the particular choices here seem very arbitrary (more or less what happened last year?!). Please see if this is worth rectifying.

Line 150 and many other places; what is the justification for selecting 6 hand-curated behaviors as ground truth? How does one generalize? For any new setting, do we first need human intuition for what the behaviors should be and then pick those for GT?

Lines 182-204: The authors might include a table or a schematic figure to explain the experimental design (line 182- 204). This will make for a convenient reading.

Line 256: the number of frames is not a round number. Some more details on what that means would be good - like 40 minutes of data with at least xx transitions into each behavior.

Line 272: but how sure are the authors that the human annotated GT is actually good? That is, could the misclassification really be a problem of mistakes by humans? If not, how to show that?

Line 307: human-interpretable seems to be a bit fixated on the 6 identified states - but can one really discount states that really exist, but are not easily interpretable with our biased expectations?

Line 357: since the picking of the particular features from RF to pass through is heavily curated by the investigator, this has to be done for every setting in every experiment. Just thinking of the combinatorial explosion, it seems really difficult to scale. It would be good to discuss this.

Lines 399-404: transition probability matrix should have all the necessary info.

Line 401 onwards: sentence fragment... “Because....” is not completed.

Line 418: Is it possible to show the entire transition matrix, may be displaying the mean or median (or mean) transition probability between all states?

## Author Response

Synthesis Statement for Author (Required):

The reviewers both felt that the manuscript merits publication and had a number of comments and suggestions for ways to strengthen the work. Please address them individually.

1. The reviewers both felt that the manuscript did not sufficiently make the advance clear, nor was it clear how scalable/generalizable the work was likely to be. Both reviewers felt that the manuscript would be stronger for comparison with other approaches. They had suggestions for specific analyses, as well as ways to broaden the Discussion.

(a) discussion of B-SOID or Deepethogram, or JAABA/GentleBoost algorithm (Branson lab)

Response: Thank you for this suggestion. The suggested content has been added to the Discussion (Line 575-578).

(b) comparison of the results to GT beyond the initial training in novel videos? Specifically with respect to #2, the authors are encouraged to classify a small number of frames and check the accuracy. The reviewer felt that “comparison to GT data not in the training set is essential.”

Response: We appreciate reviewers’ suggestion. We have applied the RF model to classify video frames that were not used to train RF model. The results have been added to the manuscript (Fig 3F; Results Lines 311-313).

(c) discussion of the appropriateness of the approach, and additional comparison to other HMM-based approaches. I’ll quote here from one of the reviewers who had a number of good suggestions:

“The problem that the authors are studying is not that hard of a ML problem and their RF itself does a really good job, particularly, after their temporal filtering (Figure 2E). The small improvements that they see are not necessarily game-changing. The classification of three states into a single state can be discovered in the Transition Probability matrix. Further, the HMM in the first step won’t be that helpful because HMM states are too short-lived. The authors can check this by plotting the duration in each state, which are likely to be very short. If true, comparison to hierarchical versions of HMM or HHMM like employed in Bhandawat et. al. would be important. Alternatively, there may be short-term structure in transition probabilities of the HMM that corresponds to each human defined 6 states - particularly in HMM with many states. You might observe that only some of the state transitions are present”

Response: we appreciate the reviewer’s comments. As expected, the HMM states are shorter than RF states (data not shown). Regarding HMM state transitions: self-transitions dominate, and the state transition matrixes have been added to the manuscript (Fig. 4C, E; Fig. 4-2B; Results Line 339-341). For the comparison with other HMM-based approaches, we utilized and compared several HMM-based model extensions (https://github.com/lindermanlab/ssm), including auto-regressive HMMs (ARHMM), Hidden Semi-Markov Models (HSMM), and Hierarchical HMM. The current HMM (hmm.GaussianHMM) showed the most useful outputs in our experimental conditions (especially since we wanted to limit the number of states in the HMM). We now note that we tried multiple HMM variants in the Methods (Lines 233-235).

(d) consideration of how other labs have leveraged HMMs. Again, quoting from a reviewer: “I am sure the authors know this and may not have meant it but it seems that they make a big deal about how HMM states are uninterpretable. Comparing an unsupervised model to a supervised model in this manner is not appropriate. Also, the reason to use HMMs is to discover things in the data that are not that obvious to humans - like speed trends etc.”

Response: We appreciate the reviewers’ comments. Instead of using “interpretability”, we changed this narrative of HMM in the manuscript to reflect the framework of a researcher-generated hypothesis. In cases where a researcher seeks to interpret behavior in the context of an experiment designed to evaluate pre-defined “behaviors” (i.e. an ethogram) with the goal of testing a hypothesis, HMMs are not always a great match. Throughout the manuscript we have limited the number of times we use the word “interpretable,” instead using “hypothesis-driven” or similar (e.g., Lines 337-339, 341-347).

2. There were three additional questions about Methods

As a human annotator, a starkly evident feature for many of the different states is the distance to the petri dish, followed by the animal’s orientation to it. Is it then surprising that the distance is a key feature (see Figure 2)? Another way of framing this is: in an arbitrary behavioral situation, what if we didn’t know what the key feature was in an intuitive way?

Response: We appreciate the reviewer’s insightful question. In this workflow, the five behavioral features (distance to petri dish, head orientations, nose/body velocities) were chosen for two reasons:

1) The five measurements, combined with the centroid position of the tracked petri dish, can be used to mainly reproduce the location and orientation of the head and body (though not fully) in the arena. This reduction from 24 datapoints (12 labels, 2 positions/label) to 5 also dramatically reduced the computational cost to train models. For example, the HMM model took less than 10 minutes to classify six states using five behavioral features. In contrast, it took over 10 hours to classify the same number of HMM states using raw DLC tracking data of 12 labels (24 features).

2) This approach minimized the effects of a moving petri dish. During experiments, animals often bumped the petri dish from its initial location (sometimes by several cm). Because the position of the petri dish was tracked, all measurements were robust to these slight changes.

In the end, it is true that the choice to compress the positional information into a minimal set of features affected the outcomes (as it pertains to the key features). In the context of this hypothesis-driven experimental setup, where there was a deliberate choice to place a chemosensory target in the open rectangular arena (right on the figures) opposite to an opaque (“safe”) zone (left on the figures), one would expect that if we supplied only the x-y positions of all features to the ML models, the x-positions of the nose and/or body would become dominant. In an arbitrary behavioral situation, we agree this choice might be limiting (Line 281-285).

Why is one of the states called “sniffing odor”, when there is no information with respect to the animal sampling the odor? It would be more appropriate to call it “Interacting with or investigating odor”

Response: We appreciate this suggestion. The “Sniffing odor” state has been renamed with “Investigating-odor”.

How did the authors deal with occlusion of body parts, when for example, the animal is grooming? How did the authors deal with frames where points were not reliably tracked?

Response: To deal with low-confidence tracking data, we first trained the DeepLabCut model to reach a high accuracy that the test error (difference between the manual-label and DLC label) was less than 5 pixels. Second, we applied a rolling median filter to smooth tracking data, which dramatically decreases the impact of spurious bad tracking (and swapped labels, etc). Third, labeled videos created by DLC were manually spot-checked prior to finalization (Line 137-139).

3. The reviewers had general comments on the figures:

The reviewers found the quantification & visualization lacking, particularly the bar plots in Figures 5-7. The authors are *strongly* encouraged to consider estimation statistics / visualization per e.g. “Moving beyond p-values: data analysis with estimation graphics” by Adam Claridge-Chang et. al. to provide a more accurate picture of their data.

Response: We understand this comment, but in all these cases we plot each individual data point, mean, and standard deviation. The individual comparisons made are intended to provide comparisons to manual, ethogram-based behavioral analysis. We agree that the number of comparisons and plots is quite large, but erred on the side of completeness.

4. As well as specific comments on the figures:

In Figure 2A, midpoint of the two ears are being plotted and it is not actually the y-axis. Correct? This was a little confusing. Marking the box as in Figure 1 would be helpful.

Response: We have remade all such plotting and added the scales (pixels) in the x- and y-axis.

The data presented in Fig 5-7 are very detailed; it might be good to conclude with a summary figure, collecting the major findings from figs 5-7.

Response: Producing a summary diagram from such a diverse set of results is quite challenging. We hope that by providing the additional experimental design figure (Fig. 2), and including the LDA analysis panels prominently in these figures (now Figs. 6-8), the readers can appreciate that the workflow identifies unique behavior sequences that reflect the chemosensory stimuli in the environment.

Figure 5-1: units in 0.25 second is not intuitive.

Response: Regarding the units of 0.25s/column: For the behavioral sequence heatmaps, the 0.25 s/column density reflects the data compression we applied, which replaced the most frequent element (RF state, HMM state) and the median of the distance metric (for reHMM+). 60 FPS / 15 frames per bin equals 0.25 second time bins. This is noted in Methods (Lines 245-248).

5. Finally, the reviewers had specific comments on the text:

The acronyms employed AP etc are not intuitive. Maybe three letter acronyms like the one authors use for Exploration would be easier to keep track of

Response: Thanks for this helpful suggestion. All ethogram states have been renamed to three-letter acronyms.

More information on the LDA is necessary

Response: more information about the LDA has been added to the Methods section (Line 254-262).

Line 7 and throughout: Is RF really the best supervised model for classifying behaviors (well, it’s really for poses to be precise)? It’s not clear why the emphasis is so much on this one, and justifying it might be good.

Response: In our behavioral experiment, we tested the performances of several supervised ML models in classifying mouse risk assessment behavior using another training dataset (Data not shown). The RF model reached the highest accuracy (0.9802), compared with K-nearest neighbors (0.9595), Support vector machine (0.9587), and Logistic Regression (0.9067) (Line 288-291). To be more accurate, we appreciate this comment and revised the narrative of the RF model in the manuscript (e.g., line 55-56, 291-293).

Line 26 (and throughout): HMMs are not really classifiers - they are state models.

Response: Agreed. We have changed the description of HMMs in the manuscript.

Lines 42/43: It will be fair to also cite LEAP by Pereira et al here (Nat Meth 16:117). The authors used the outputs of DLC, but in principle, these methods would work for other pose estimation methods such as LEAP. Also, Mathis et al is incorrectly double cited - please correct this and any other reference/citation issues.

Response: The suggested reference has been added, and the incorrect citation has been corrected (Line 43).

Line 54: Siddiqui and Medioni looks more like a pose-estimation rather than behavior retrieval algorithm? Please clarify.

Response: Apologies for these incorrect citations. The correct references have been added to the manuscript (Line 54) .

Lines 62/63: The authors make an important point about the differences in classifying behaviors based on frames without taking into account sequences, vs methods to do this taking into account temporal information. But pitching it as RF vs HMM seems odd - there are many other sorts of classifiers of poses based on single frames than RFs. Perhaps it will help the reader to get this point better by pointing out that RF is one of many classifiers based on single frames? Bottom line: it’s not really RF vs HMM, but static classifiers vs ones based on dynamics (ideally with different temporal scales).

Response: Thanks for this suggestion. This description has been revised (Line 332-334).

Line 72: all of these applications are vast, so hard to cite specific ones - but the particular choices here seem very arbitrary (more or less what happened last year?!). Please see if this is worth rectifying.

Response: Thank you so much for this good suggestion. Two reviews published in 2022 would be good references cited here (Line 73):

Glennie, Richard, et al. “Hidden Markov models: Pitfalls and opportunities in ecology.” Methods in Ecology and Evolution (2022).

Ruiz-Suarez, Sofia, Vianey Leos-Barajas, and Juan Manuel Morales. “Hidden Markov and Semi-Markov Models When and Why are These Models Useful for Classifying States in Time Series Data?.” Journal of Agricultural, Biological and Environmental Statistics 27.2 (2022): 339-363.

Line 150 and many other places; what is the justification for selecting 6 hand-curated behaviors as ground truth? How does one generalize? For any new setting, do we first need human intuition for what the behaviors should be and then pick those for GT?

Response: We appreciate this fundamental question. The ground truth (6 behavior states) was defined based on the experiment’s objective of investigating the mouse risk assessment behavior and previous reports (Dielenberg et al. 2001, PMID: 11801285) (Line 154-155). This reference has been added to the manuscript. In our opinion, many behavioral experiments benefit from human intuition at multiple levels, and ultimately making conclusions requires the experimenters to intrerpret results in the context of a hypothesis. By pre-defining an ethogram in this context, we introduce this pre-classification, rather than doing so post-classification. There are of course benefits to fully unsupervised approaches (i.e. the identification of poses/sequences outside the ethogram framework), but this capacity comes along with difficulties, for example the presence of many seemingly arbitrarily divided states (i.e. duplicates with little obvious differences (e.g. Fig 4-1, HMM(11)_4, HMM(11)_6).

Overall, as mentioned in the two reviews now cited above (Glennie et al. 2022; Ruiz-Suarez et al. 2022), unsupervised states may not map to specific behaviors, limiting their utility in many cases.

Lines 182-204: The authors might include a table or a schematic figure to explain the experimental design (line 182- 204). This will make for a convenient reading.

Response: We appreciate this constructive suggestion. A figure illustrating the experimental design has been added to the manuscript (Figure 2).

Line 256: the number of frames is not a round number. Some more details on what that means would be good - like 40 minutes of data with at least xx transitions into each behavior.

Response: In this study, the video was recorded at 60 FPS. The beginning and end of the recording process were manually controlled, and the ending point was not at regular intervals of 1 second. Thus, the total frames of one video were not the multiple of 60.

Line 272: but how sure are the authors that the human annotated GT is actually good? That is, could the misclassification really be a problem of mistakes by humans? If not, how to show that?

Response: The GT data were manually annotated on a full, frame-by-frame basis across 12 videos without reference to the DeepLabCut tracking data. The results, plotted after applying DLC tracking (now Fig. 3A) show that the positions, head directions, and velocities of the animals in these conditions were consistent with the ethogram definitions (Fig. 1D). Note that a handful of points in Fig. 3A appear discordant, which reflects a very small number of frames for which the DLC tracking data was erroneous, even after applying the rolling median filter (this was confirmed by manual inspection).

Line 307: human-interpretable seems to be a bit fixated on the 6 identified states - but can one really discount states that really exist, but are not easily interpretable with our biased expectations?

Response: As discussed above, the choice to define an ethogram is part of the experimental design. We also now clarify that “interpretable” is perhaps the wrong term (we have now emphasized hypothesis-related states). It is true that in this workflow the user is able to define the states related to their hypothesis, and that unsupervised methods might identify states outside their definition of use, and we acknowledge this in the manuscript (Line 73-74, 337-347).

Line 357: since the picking of the particular features from RF to pass through is heavily curated by the investigator, this has to be done for every setting in every experiment. Just thinking of the combinatorial explosion, it seems really difficult to scale. It would be good to discuss this.

Response: Thanks for this comment. The primary rationale for picking features for the reHMM+ model was based on the “Feature Importance” of the Random Forest model. In this study, the distance was the most informative feature for RF classification. Thus, we selected distance as feature to train the reHMM+ model. In studies omitted due to space, we also included other informative features (in addition to distance), finding limited utility. We also appreciate the reviewer’s point about the scenario of combinatorial explosion. In the future we plan to incorporate Explainable Artificial Intelligence (XAI) techniques into our workflow, which we hope will improve the transparency and explainability of the ML models, as well as assist in selecting features for the reHMM+ model. We now mention this in the Discussion (Line 638-643).

Lines 399-404: transition probability matrix should have all the necessary info.

Response: The transition matrix of each animal in response to each odorant was summarized and added to supplementary materials (e.g., Fig 4C, 4E, 4-2B, 6-2, 7-2, 8-2).

Line 401 onwards: sentence fragment... “Because....” is not completed.

Response: The sentence has been reworded.

Line 418: Is it possible to show the entire transition matrix, may be displaying the mean or median (or mean) transition probability between all states?

Response: The transition matrix of each animal in response to each odorant was summarized and added to supplementary materials (e.g., Fig 4C, 4E, 4-2B, 6-2, 7-2, 8-2).
