# Supplementary figures and images for: A Layered, Hybrid Machine Learning Analytic Workflow for Mouse Risk Assessment Behavior
Source: eNeuro. 2022 Jan 6;10(1):ENEURO.0335-22.2022. doi: 10.1523/ENEURO.0335-22.2022 (PMC9833056; doi:10.1523/ENEURO.0335-22.2022)

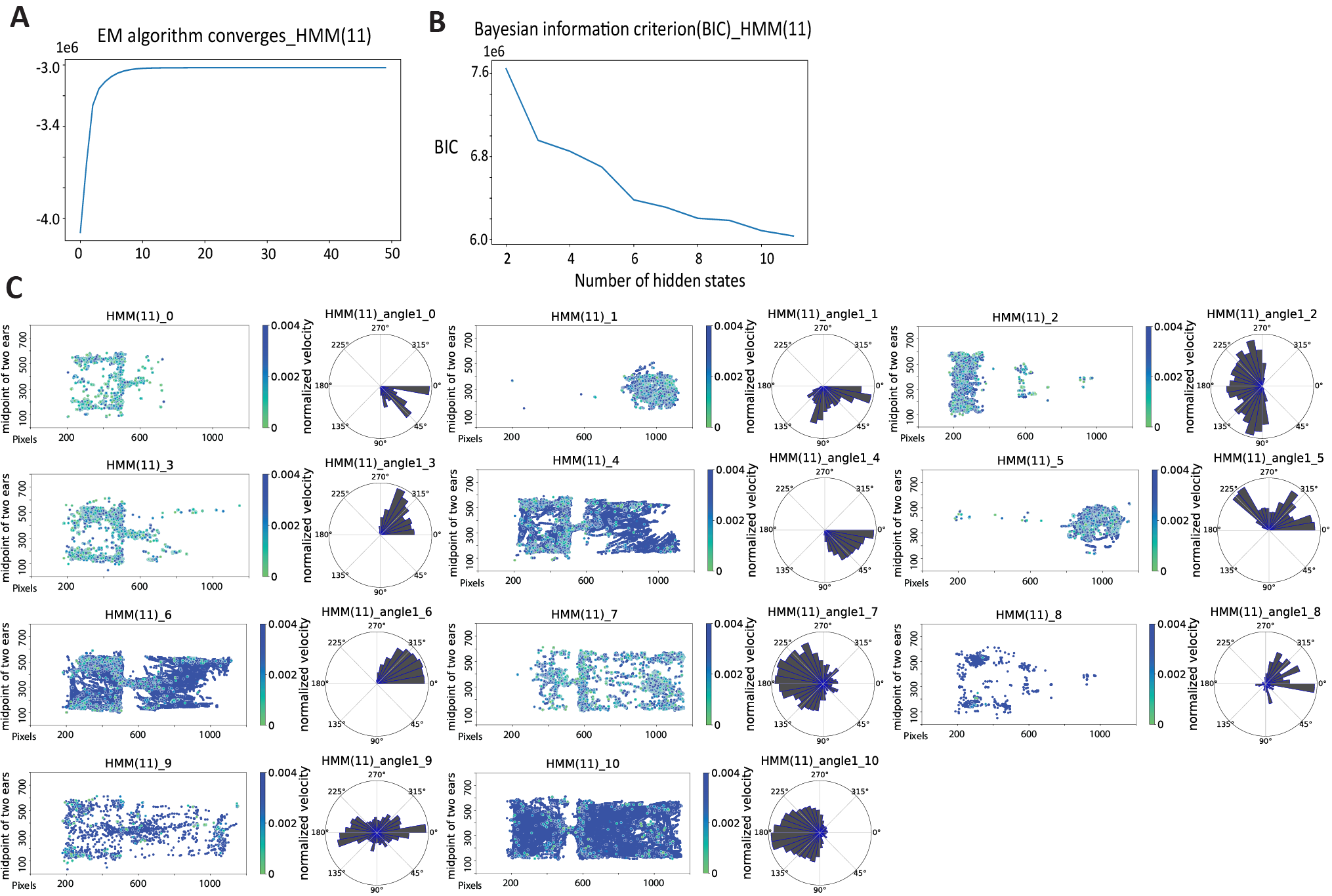

Supplement: Extended Data Figure 4-1 — Overview of hidden Markov model for 11-state classification. A, Expectation-Maximization (EM) algorithm for hidden Markov model for 11-state classification [HMM(11)] relative to the number of training iterations. B, Bayesian Information Criterion (BIC) scores of HMM(11) relative to the number of hidden states. C, Graphical representations of hidden behavioral states, as predicted by HMM(11). For each behavioral state, dots represent the midpoint of two ears, and the color represents the velocity of the body center. The right polar plot represents the angle between the head direction vector and the horizontal x-axis. Download Figure 4-1, TIF file. [file enu-eN-MNT-0335-22-s08.tif]

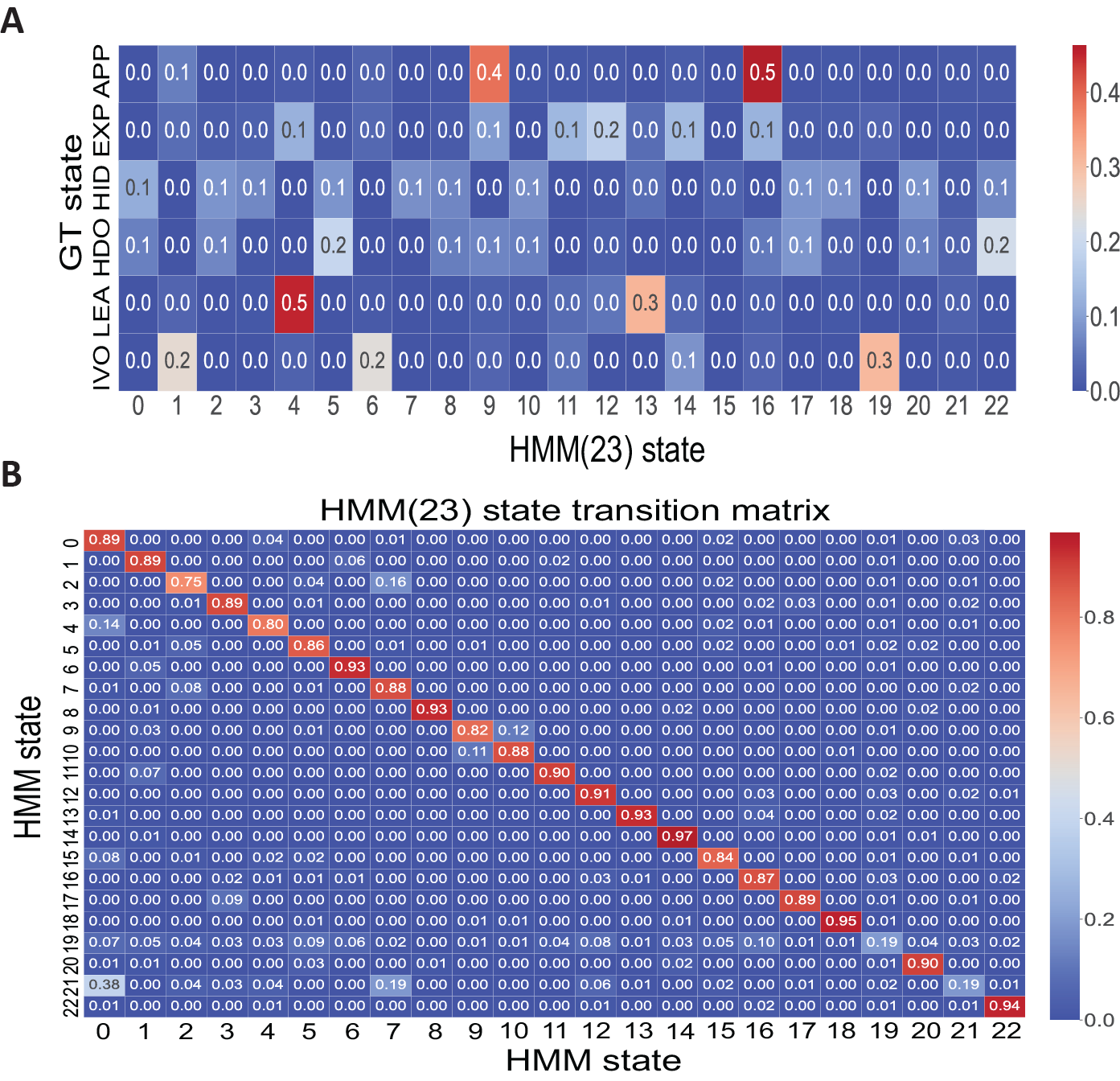

Supplement: Extended Data Figure 4-2 — Performance of the hidden Markov model for 23-state classification. A, Confusion matrix for the HMM for 23-state classification [HMM(23)] versus the GT. B, State transition matrix of hidden Markov model for 23-state classification. Download Figure 4-2, TIF file. [file enu-eN-MNT-0335-22-s03.tif]

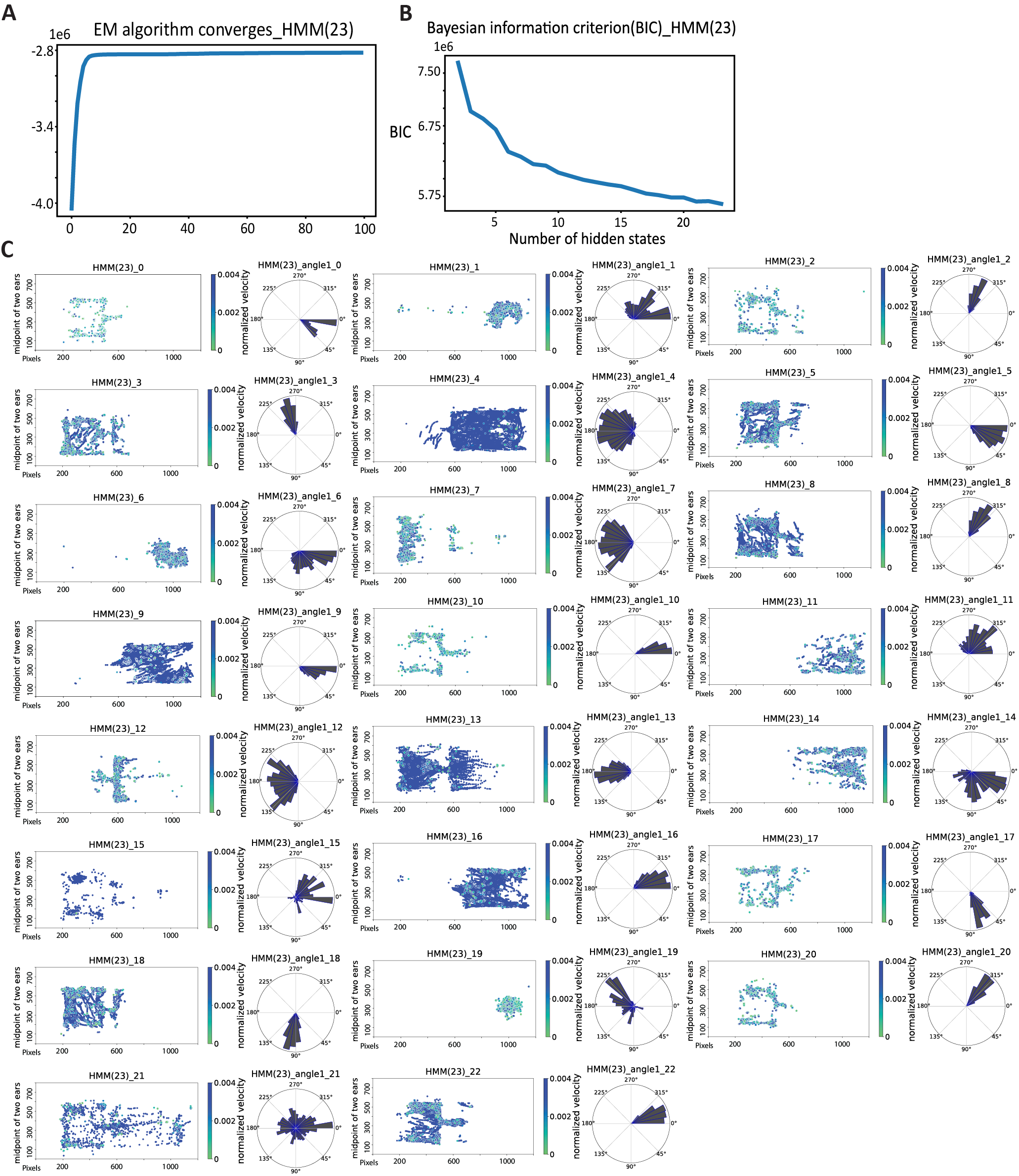

Supplement: Extended Data Figure 4-3 — Overview of hidden Markov model for 23-state classification. A, Expectation-Maximization (EM) algorithm for hidden Markov model for 23-state classification [HMM(23)] relative to the number of training iterations. B, Bayesian Information Criterion (BIC) scores of HMM(23) relative to the number of hidden states. C, Graphical representations of hidden behavioral states, as predicted by HMM(23). The dots represent the midpoint of two ears, and the color represents the velocity of the body center. The right polar plot represents the angle between the head direction vector and the horizontal x-axis. Download Figure 4-3, TIF file. [file enu-eN-MNT-0335-22-s04.tif]

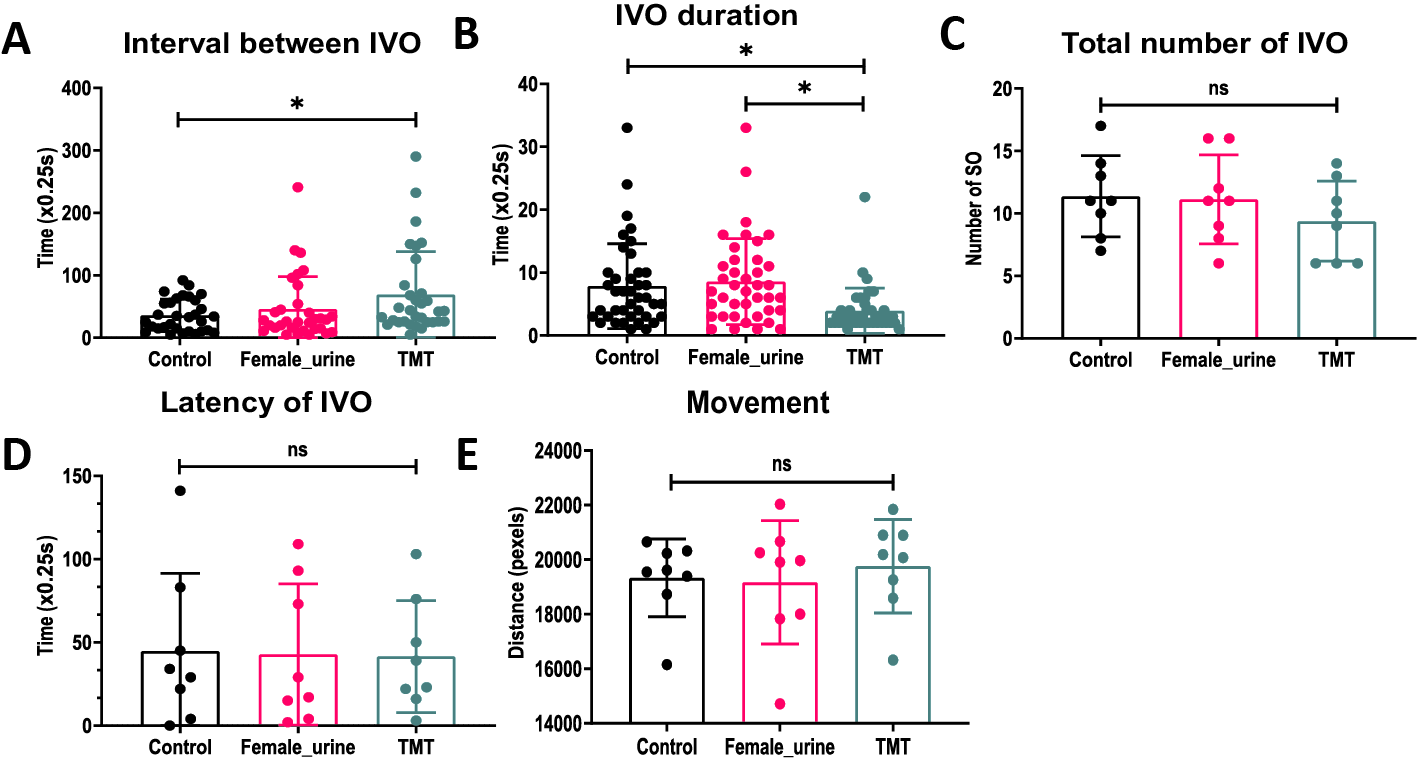

Supplement: Extended Data Figure 6-1 — Additional analysis of male mouse behavioral responses to TMT and female mouse urine. A, Interval between two consecutive IVOs. B, Duration of each IVO. C, Total number of IVO. D, Latency of the first IVO. E, Total movement distance; *p < 0.05, one-way ANOVA followed by multiple comparison’s tests. A total of 24 mice (n = 8 for each treatment group) were available for analysis. Additional data can be found in Extended Data Figure 6-1 and Extended Data 6-2. Download Figure 6-1, TIF file. [file enu-eN-MNT-0335-22-s05.tif]

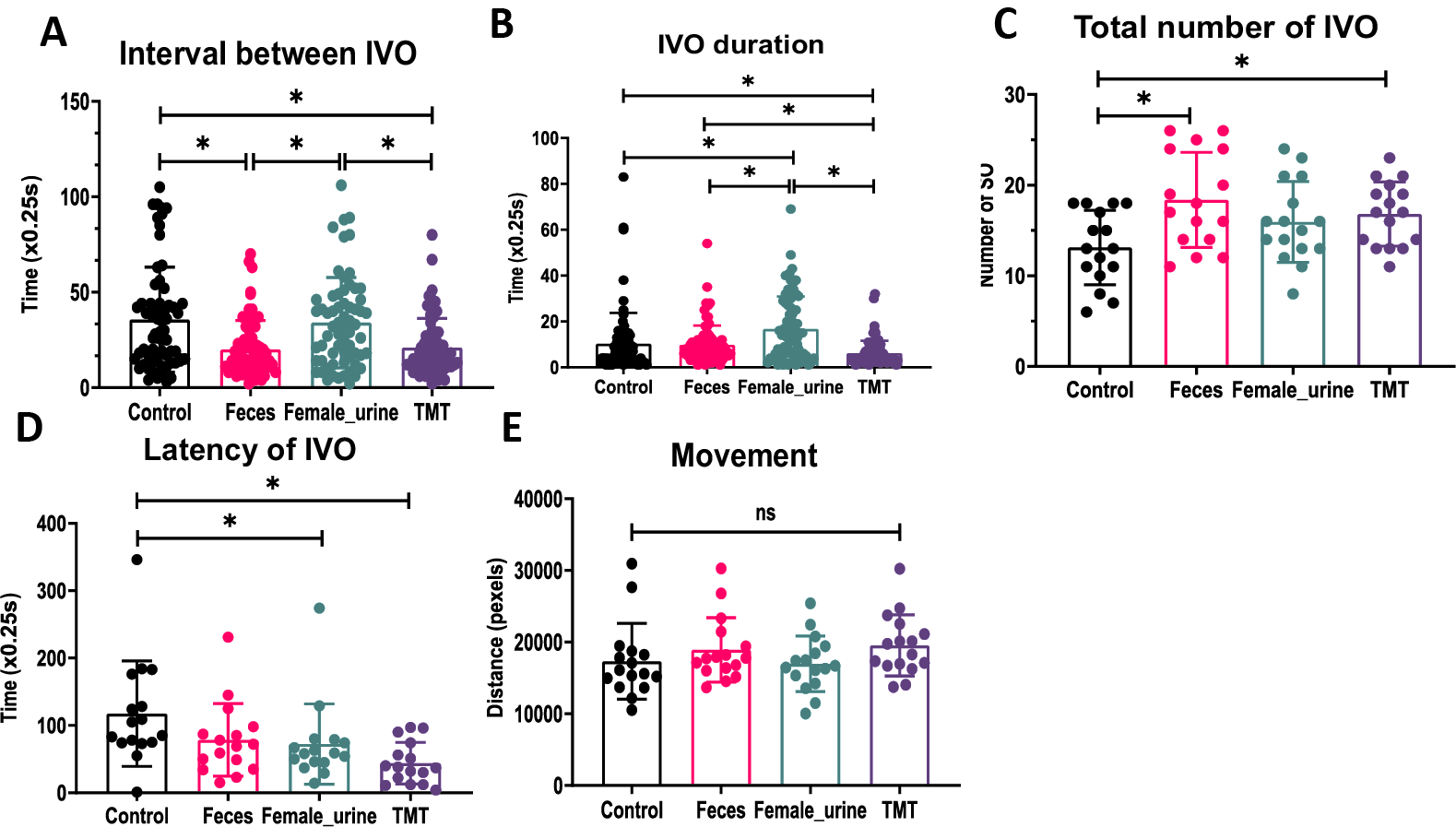

Supplement: Extended Data Figure 7-1 — Additional analysis of male mice behavioral responses to female mouse urine, snake feces, and TMT. A, Interval between two consecutive IVOs. B, Duration of each IVO. C, Total number of IVO. D, Latency of the first IVO. E, Total movement distance; *p < 0.05, one-way ANOVA followed by multiple comparison’s tests. For this experiment, 16 mice were available for analysis. Download Figure 7-1, TIF file. [file enu-eN-MNT-0335-22-s06.tif]

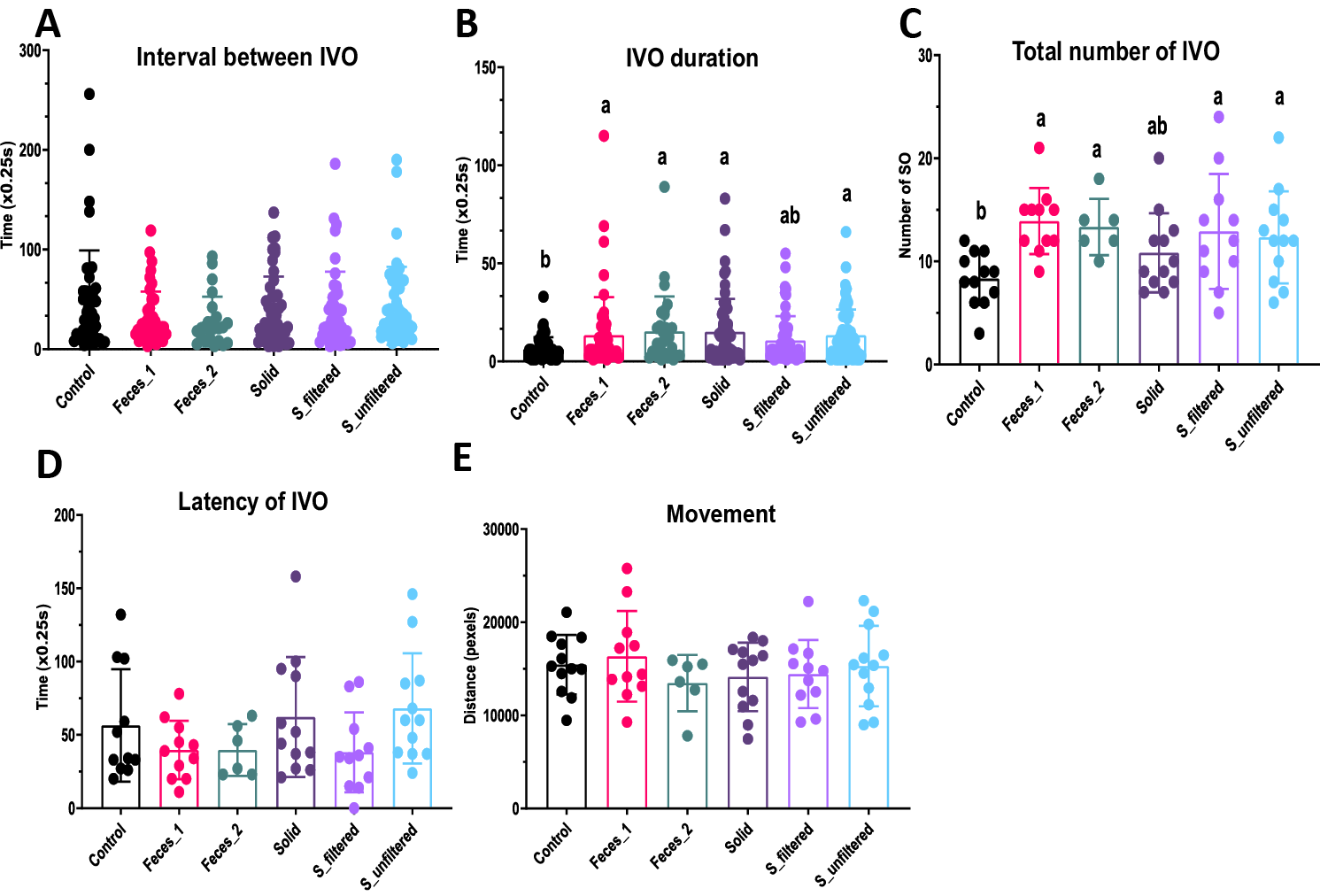

Supplement: Extended Data Figure 8-1 — Additional analysis of male mouse behavioral responses to snake feces and its extracts. A, Interval between two consecutive IVOs. B, Duration of each IVO. C, Total number of IVO. D, Latency of the first IVO. E, Total movement distance; *p < 0.05, one-way ANOVA followed by multiple comparison’s tests. For this experiment, six mice were available for analysis. Download Figure 8-1, TIF file. [file enu-eN-MNT-0335-22-s07.tif]
